# Genomic trajectories of a near-extinction event in the Chatham Island black robin

**DOI:** 10.1186/s12864-022-08963-1

**Published:** 2022-11-10

**Authors:** Johanna von Seth, Tom van der Valk, Edana Lord, Hanna Sigeman, Remi-André Olsen, Michael Knapp, Olga Kardailsky, Fiona Robertson, Marie Hale, Dave Houston, Euan Kennedy, Love Dalén, Karin Norén, Melanie Massaro, Bruce C. Robertson, Nicolas Dussex

**Affiliations:** 1 Centre for Palaeogenetics, Svante Arrhenius Väg 20C, 106 91 Stockholm, Sweden; 2 Department of Bioinformatics and Genetics, Swedish Museum of Natural History, Stockholm, Sweden; 3 Department of Zoology, Stockholm University, 106 91 Stockholm, Sweden; 4 Department of Biology, Lund University, Ecology Building, 223 62 Lund, Sweden; 5 Ecology and Genetics Research Unit, University of Oulu, 90014 Oulu, Finland; 6 Department of Biochemistry and Biophysics, Science for Life Laboratory, Stockholm University, 17121 Solna, Sweden; 7 Department of Anatomy, University of Otago, Dunedin, 9054 New Zealand; 8 Coastal People Southern Skies Centre of Research Excellence, University of Otago, PO Box 56, Dunedin, 9054 Aotearoa New Zealand; 9 Department of Zoology, University of Otago, Dunedin, 9054 New Zealand; 10 School of Biological Sciences, University of Canterbury, Christchurch, 8140 New Zealand; 11 Department of Conservation, Biodiversity Group, Auckland, New Zealand; 12 Department of Conservation, Science and Capability, Christchurch, New Zealand; 13 School of Agricultural, Environmental and Veterinary Sciences and Gulbali Institute, Charles Sturt University, PO Box 789, Albury, NSW Australia

**Keywords:** Mutational load, Inbreeding, Genomics, Bottleneck, Near-extinction, Translocation

## Abstract

**Background:**

Understanding the micro-­evolutionary response of populations to demographic declines is a major goal in evolutionary and conservation biology. In small populations, genetic drift can lead to an accumulation of deleterious mutations, which will increase the risk of extinction. However, demographic recovery can still occur after extreme declines, suggesting that natural selection may purge deleterious mutations, even in extremely small populations. The Chatham Island black robin (*Petroica traversi)* is arguably the most inbred bird species in the world. It avoided imminent extinction in the early 1980s and after a remarkable recovery from a single pair, a second population was established and the two extant populations have evolved in complete isolation since then. Here, we analysed 52 modern and historical genomes to examine the genomic consequences of this extreme bottleneck and the subsequent translocation.

**Results:**

We found evidence for two-fold decline in heterozygosity and three- to four-fold increase in inbreeding in modern genomes. Moreover, there was partial support for temporal reduction in total load for detrimental variation. In contrast, compared to historical genomes, modern genomes showed a significantly higher realised load, reflecting the temporal increase in inbreeding. Furthermore, the translocation induced only small changes in the frequency of deleterious alleles, with the majority of detrimental variation being shared between the two populations.

**Conclusion:**

Our results highlight the dynamics of mutational load in a species that recovered from the brink of extinction, and show rather limited temporal changes in mutational load. We hypothesise that ancestral purging may have been facilitated by population fragmentation and isolation on several islands for thousands of generations and may have already reduced much of the highly deleterious load well before human arrival and introduction of pests to the archipelago. The majority of fixed deleterious variation was shared between the modern populations, but translocation of individuals with low mutational load could possibly mitigate further fixation of high-frequency deleterious variation.

**Supplementary Information:**

The online version contains supplementary material available at 10.1186/s12864-022-08963-1.

## Background

In the current biodiversity crisis, an increasing number of species survive in ever smaller and fragmented populations [1–3]. These species risk being trapped in an extinction vortex owing to a combination of demographic processes and genetic threats such as inbreeding and accumulation of harmful mutations (i.e., mutational load [4, 5]).

Population genetic theory suggests two different outcomes for mutational load in small populations. Under one scenario, small populations will accumulate deleterious mutations as a consequence of strong genetic drift and reduced efficacy of purifying selection [6], leading to an increased extinction risk via ‘mutational meltdown’ [4]. A number of empirical studies on species that have experienced sudden and severe declines support this scenario (e.g., crested ibis, *Nipponia nippon* [7]; Grauer’s gorillas, *Gorilla beringei graueri* [8]). An alternative outcome is that purifying selection reduces mutational load under a specific set of circumstances. For instance, gradual population decline or long-term population isolation combined with small population size, as well as increased selection against recessive or partially-recessive detrimental alleles owing to increased homozygosity, can induce purging of mutational load [9–11]. An increasing body of empirical genomic studies support this alternative scenario of purging of mutational load in the wild (e.g., mountain gorilla, *G. b. beringei* [8]; kākāpō, *Strigops habroptilus* [12]; Indian tiger, ​​*Panthera tigris tigris* [13]; Montezuma quail, *Cyrtonyx montezumae* [14]; Alpine Ibex, *Capra ibex* [15]; rattlesnake, *Sistrurus catenatus* [16]; Channel Island fox, *Urocyon littoralis* [17]).

The amount of mutational load will also be affected by species management strategies, including captive breeding or the intentional movement of individuals from one area to another (i.e., translocations [18, 19]), as these approaches will directly impact effective population size (N_e_) [20]. For instance, it has been suggested that artificially inducing inbred matings could reduce mutational load [10]. Strong support for the success of such matings is however still lacking (e.g., Speke's gazelle, *Gazella spekei* [21, 22]). In contrast, subdividing populations during translocations or when creating ‘insurance populations’ may lead to parallel evolution (i.e., differential fixation) of this load, but also presents a high risk of increasing drift and fixation of deleterious alleles, especially in extremely small populations [23]. As many of the most threatened species survive as small and inbred populations, hands-on conservation programmes have been essential to their protection. To manage the risk of localised threats to these species, ‘insurance populations’ are often established via translocation of individuals to other sites [18, 24, 25]. Since translocations primarily aim to maintain genetic diversity and adaptive potential [26], for instance by reducing reproductive variance among individuals, one unintended consequence is that populations may fix or lose different detrimental and beneficial alleles through drift, which is particularly strong in small populations [27]. It is thus crucial to weigh the genetic risks and benefits of translocations [26].

A species that is in urgent need of genetic management is the critically endangered Chatham Island black robin (karure, *Petroica traversi*), a passerine endemic to the Chatham Islands (Rēkohu), a small island group 800 km east of New Zealand. Over the past 200 years, the species’ range has contracted due to habitat modification and the introduction of mammalian predators such as rats (*Rattus sp*.). Between 1893 and 1976, black robins were restricted to ~ 35 birds on a small island (Tapuaenuku/Little Mangere [28]) and declined to seven birds [28–31]. The remaining birds were translocated to an adjacent island (Maung’ Rē/Mangere) in 1976, and declined to five birds before management efforts helped reverse the negative population trend [29]. After a remarkable recovery from a single breeding pair producing viable young [32], a second population was established via sequential translocations of 23 birds and 53 eggs from Maung’ Rē onto Hokorereoro/South East Island between 1983 and 1990 [28–31]. As of 2021, there were 25 birds on Maung’ Rē and 273 on Hokorereoro.

The black robin is an ideal model species to study micro-evolution in small populations and to obtain an understanding of the genomic impacts of conservation actions. The two extant populations are descendants of the same breeding pair and have been separated for 31 breeding seasons since the last translocation event in 1990 [29]. While it is arguably the most inbred bird species in the world [33, 34] and is affected by inbreeding depression [35], high survival and reproductive performance suggest that purging of mutational load may have occurred in the evolutionary history of the species and/or during the bottleneck spanning 1893 to 1976 [28, 33]. Interestingly, pedigree data showed improved survival for fledglings with highly inbred mothers, but not with highly inbred fathers [33], suggesting that some reduction of load may indeed have occurred, through a mechanism similar to purging [33]. In contrast, one previous study found no support for purging of genetic load in black robin [35] and that some maladaptive traits with a genetic basis (i.e., rim-laying of eggs) remain in the populations (estimated at ~ 20% [32]). This suggests that while some purging may have occurred, some deleterious variation may have also been retained [32].

Generating complete genomes for non-model and endangered species has been instrumental in furthering our understanding of the genomic consequences of severe population declines and aiding conservation efforts of these taxa [36, 37]. Several recent studies have also used temporally-spaced complete genomes from isolated populations to directly measure change in genomic parameters due to population decline [7, 8, 12, 38–40]. It should be thus possible to test the competing hypothesis of accumulation or purging of mutational load in black robins by comparing changes in the frequency of deleterious mutations through time and between populations.

Here, we analysed 52 Chatham Island black robin genomes sampled over the past 150 years, including 17 historical pre-bottleneck genomes, to test whether the near-extinction event led to accumulation or purging of mutational load in one of the most inbred bird species in the world. We also assess whether translocations resulted in differential fixation of deleterious variants in the two extant populations. Examining the genomic consequences of near-extinction in Chatham Island black robin provides important insights into the genomics and evolutionary dynamics of severely bottlenecked populations.

## Results

### De-novo genome assembly and data mapping statistics

We sequenced a Chromium linked-read library and generated a *de-novo* assembly comprising 17,752 scaffolds with a contig N50 of 85.05 Kb, scaffold N50 of 14.1 Mb, L50 of 25 and a total genome size of 1.07 Gb. 110 scaffolds had a size $\ge$ 1 Mb, representing 87% of the total assembly and the maximal scaffold length was 43.6 Mb. Moreover, the assembly had an intra-scaffold gap % (*i.e*., %N bases) of 4.68%. From the BUSCO analysis run using the avian (odb10) dataset, the assembly showed a high degree of gene-completion with 7,776 of 8,338 (93.3%) avian genes discovered as complete and with only 88 of them discovered in duplicates (1.1%). Moreover, 2.4% genes were discovered in fragments and 4.3% missing.

**Fig. 1:**
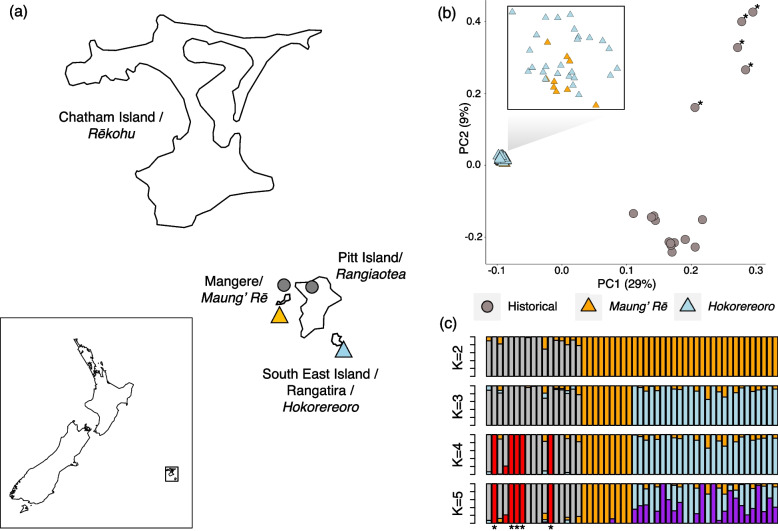
Sampling locations and population structure of black robin (*Petroica traversi*). **a** Map depicting New Zealand and the Chatham Islands with sampling locations. Circles and triangles represent historical and modern samples, respectively. Although the exact sampling location of historical specimens is sometimes uncertain and incomplete (Table S1), the origin of samples with more accurate sampling information is shown on the map. **b** Principal Component Analysis (PCA). **(c)** Admixture plot for K = 2–5. Historical specimens with inconsistent labelling and clustering are shown in the PCA and Admixture plot with asterisks

In addition, we re-sequenced 52 genomes (depth of coverage range: 3-24X; average = 13X; Table S1), of which 17 were historical genomes predating the bottleneck and 35 modern genomes from Maung’ Rē and Hokorereoro islands (Fig. 1a), which were used for population structure and demographic analyses. Out of these, 42 had high depth of coverage (range: 11-24X, average = 15X; Table S1) and were used to estimate inbreeding, heterozygosity, and mutational load. The percentage of the genome covered at 5X (i.e., our minimum depth of coverage filter for variant calling) was lower in historical (average: 77.2%) compared to modern ones (average: 85.7%) but high enough to retain a total 2,731,301 SNPs sites across all genomes.

### Population structure and past demography

Principal component and ADMIXTURE analysis based on all autosomal single-nucleotide polymorphisms revealed high heterogeneity among historical genomes but supported two main clusters (Fig. 1b,c, S4). However, sampling location information for these museum specimens was often missing or incomplete (Table S1). Moreover, the clustering of some specimens in the PCA and ADMIXTURE analyses was not consistent with their geographical origin. Conversely, samples from the two modern populations were highly homogeneous, which is consistent with the recent population history of the species.

**Fig. 2:**
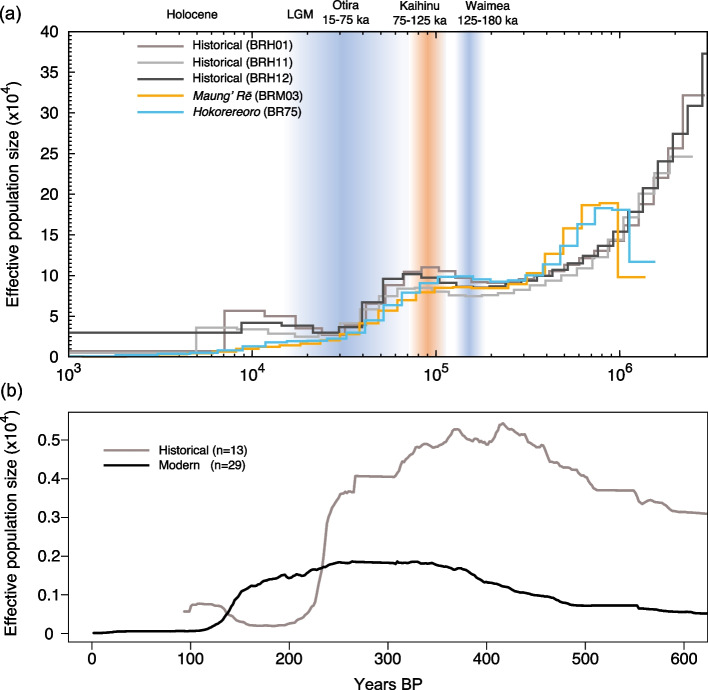
Past demography of the black robin (*Petroica traversi*). **a** Demographic history of the black robin including historical and modern birds using the PSMC approach assuming a substitution rate of 2.3×10^-9^ substitutions/site/generation and a generation time of 2 years. Each curve represents an individual genome. Approximate timing of glacial and interglacial periods are shown in blue and orange, respectively. **(b)** Demographic history over the past 600 years estimated with GONE. Maung’ Rē and Hokorereoro are grouped into a single population

The reconstruction of past demography using the PSMC approach showed a continuous decline in effective population size (N_e_) in modern genomes since ~ 100–90 thousand years Before Present (ka BP). The decline seemed to be less severe from ~ 30 ka BP onwards but continued into the mid-Holocene (i.e., < 10 ka BP; Fig. 2a, S5a,b). Moreover, while historical genomes also showed an initial decline starting some 90–80 ka BP, there was an increase in N_e_ ~ 30 ka BP. The SMC + + analysis also supported a population decline but starting slightly later ~ 75 ka BP, continuing after the end of the Last Glacial Maximum (LGM; 25–18 ka BP), and followed by a constant N_e_ over the past few centuries (Fig. S5c). Estimation of recent fluctuations in N_e_ using the linkage disequilibrium approach implemented in GONE showed a decline starting ~ 150 and ~ 300 years BP in modern and historical birds, respectively (Fig. 2b).

### Heterozygosity and inbreeding

**Fig. 3:**
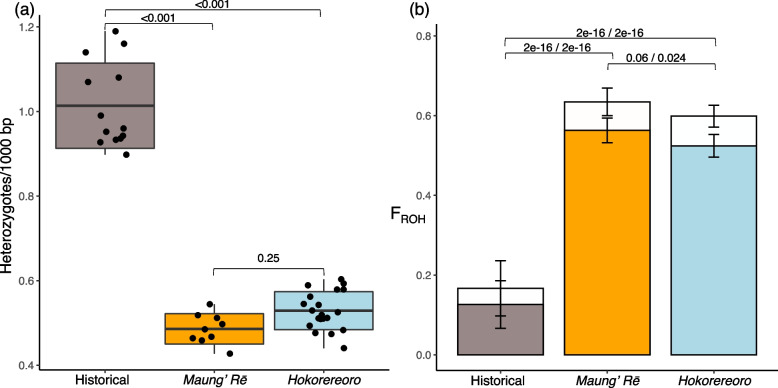
Heterozygosity and inbreeding estimates for black robin (*Petroica traversi*). **a** Genome-wide autosomal heterozygosity (heterozygous sites / 1,000 bp). Horizontal lines within boxplots depict the mean, bounds of boxes represent the standard deviation and vertical bars represent minima and maxima. **b** Inbreeding coefficients estimated using ROH (F_ROH_). Open bars show the total proportion of the genome in ROH $\ge$ 100 kb and solid bars show proportions in ROH $\ge$ 2 Mb with respective p-values separated by ‘/’. Bars extending from the mean values represent the standard deviation

We found significant differences in heterozygosity (number of heterozygous sites per 1,000 bp) and inbreeding (measured as the fraction of each genome in runs of homozygosity; F_ROH_) between historical specimens and the two extant populations (Fig. 3; Table S4).

Heterozygosity was twice as high in historical specimens with an average of 1 heterozygote site per 1,000 bp compared to the extant populations that had 0.48 and 0.53 heterozygous sites per 1,000 bp for Maung’ Rē and Hokorereoro, respectively. Genome-wide estimates of nucleotide diversity ($\pi$) also showed a ~ 1.8 fold reduction in nucleotide diversity (Fig. S6).

Inbreeding coefficients (F_ROH_) indicated a three- to four-fold increase in inbreeding for both short ROH $\ge$ 100 kb (F_ROH-Hist._ = 0.17; F_ROH-Maung._ = 0.63; F_ROH-Hoko._ = 0.59; Tukey HSD, *p* = 0.06—2e-16) and long ROH $\ge$ 2 Mb (F_ROH-Hist._ = 0.13; F_ROH-Maung._ = 0.56; F_ROH-Hoko._ = 0.52; Tukey HSD, *p* = 0.024—2e-16; Fig. 3). Moreover, frequency distribution of ROH indicated that while modern genomes showed a large proportion of long ROH $\ge$ 2 Mb (max length = 43.6 Mb), there was also evidence for a few long ROH in historical genomes (max length = 31.1 Mb; Fig. S7).

When considering the modern populations, there was only a significant difference in F_ROH_ for ROH $\ge$ 2 Mb (*p* = 0.024). These differences held when using alternative parameters to estimate F_ROH_ (Fig. S8).

### Mutational load

**Fig. 4:**
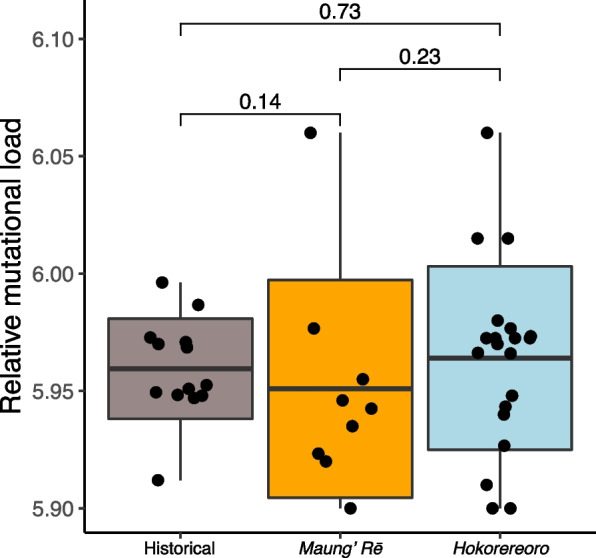
Estimates of mutational load based on GERP scores for black robin (*Petroica traversi*). Individual relative mutational load is based on GERP scores > 5.88 (i.e., derived sites in the top 1% highest GERP score values and considered as highly deleterious). Horizontal lines within boxplots depict the mean, bounds of boxes represent the standard deviation and vertical bars represent minima and maxima

We used conserved genomic regions, as estimated by using GERP scores, to estimate individual relative mutational load, and found no significant difference among populations at sites within the top 1% highest GERP scores, where mutations are likely to be damaging (here represented by GERP score > 5.88, Fig. 4). At sites with relatively low GERP scores (where mutations are assumed to have low impact on fitness), historical genomes had a higher number of derived alleles compared to the two modern populations on Maung’ Rē and Hokorereoro (Fig. S9-10).

**Fig. 5:**
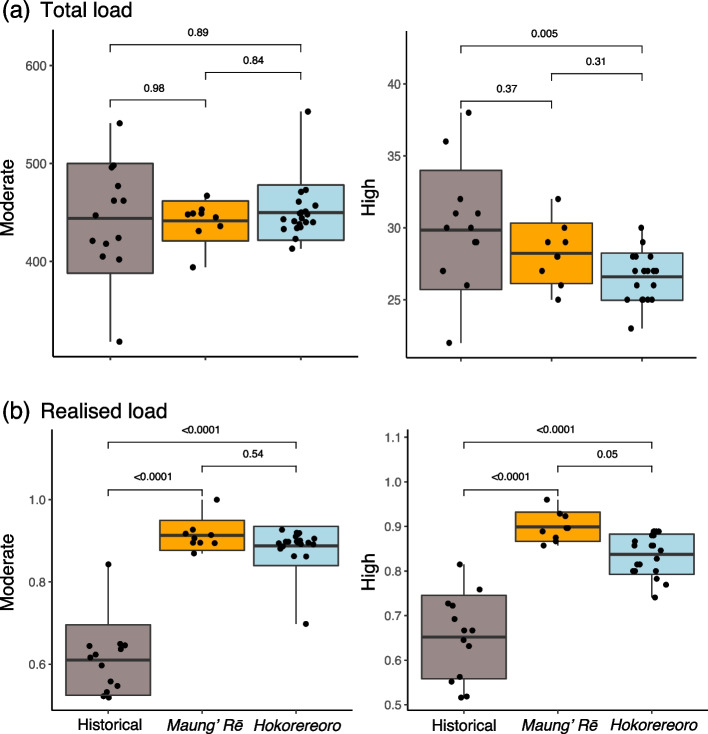
Estimates of mutational load in coding regions for black robin (*Petroica traversi*). **a** Total load. **b** Realised load. Horizontal lines within boxplots depict the mean, bounds of boxes represent the standard deviation and vertical bars represent minima and maxima

We also estimated individual load in coding regions for two categories of variants (i.e., Moderate and High impact) using SNPeff. For each category, we calculated the total load as the sum of the number of variants and the realised load as the proportion of deleterious variants expressed (i.e., in homozygous state) in an individual genome (see Methods section). We found no difference in total load for Moderate impact variants (i.e., missense alleles). However, the historical genomes had higher total load for High impact variants (i.e., loss of function alleles), but this difference was only significant relative to the modern samples on Hokorereoro (Fig. 5a, S11; Table S5). Both modern populations had significantly fewer Moderate and High impact variants in heterozygous state (Fig. S12), but significantly higher realised load (i.e., homozygous Moderate and High impact variants; Fig. 5b) compared to historical genomes, with Hokorereoro showing lower load than Maung’ Rē.

**Fig. 6:**
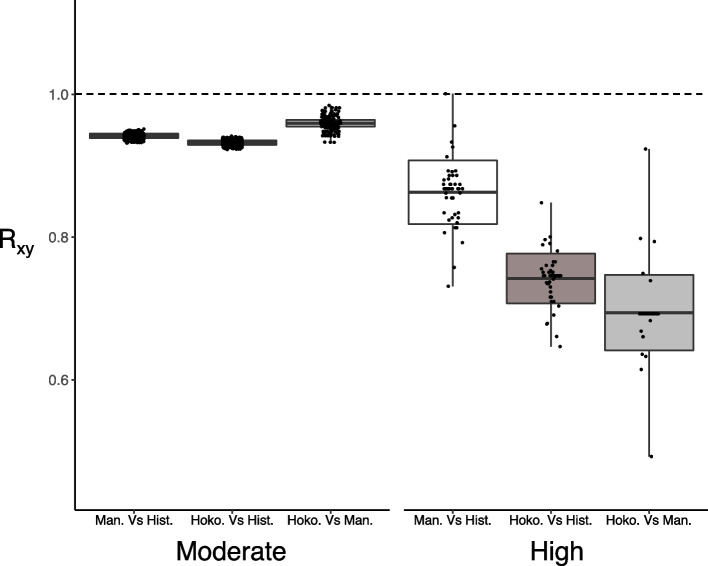
R_xy_ of derived alleles for Moderate and High impact variants for black robin (*Petroica traversi*). R_xy_ < 1 indicates a relative frequency decrease in population x vs y, for a given variant category (Man. = Maung’ Rē, Hoko. = Hokorereoro, Hist. = Historical). R_xy_ distributions are based on Jack-knifing across chromosomes

When estimating the relative frequency decrease of variants for each population pair (i.e., R_xy_), we found a reduction in High impact variants for both Maung’ Rē and Hokorereoro relative to historical genomes (Fig. 6). It is worth noting, though, that the temporal decrease between historical genomes and the surviving Maung’ Rē population is close to a value of 1, which corresponds to no change between populations. Contrary to the total load estimation, R_xy_ indicated a weak reduction in Moderate impact variants as well.

When examining load in the ‘*two populations’* dataset comprising modern birds only, we identified a total of 47 High impact variants, with 44 in Maung’ Rē (out of which 25 are fixed) and 46 in Hokorereoro (out of which 23 are fixed; Table S6). There were a total of 43 High impact variants shared between populations, and one and three unique to Maung’ Rē and Hokorereoro, respectively. Moreover, among these shared variants, 23 were fixed in both populations. A total of 10 variants increased and another 11 decreased in frequency after translocation of birds to Hokorereoro. We also identified a total of 552 Moderate impact variants, with 514 shared between populations, as well as 34 and 4 unique to Maung’ Rē and Hokorereoro, respectively. Moreover, there were 320 and 290 of these moderate variants fixed in Maung’ Rē and Hokorereoro, respectively, and 208 variants fixed in both populations (Table S6).

The Gene Ontology analysis for genes containing High impact variants in the two modern populations identified genes associated with various biological processes, including cellular processes (e.g., DNA repair/replication), biological regulation (e.g., phospholipid-transporting), and localization (e.g., anion exchange; Table S7). There was no significant overrepresentation of any biological process.

## Discussion

Here, we examined the genome-wide consequences of near-extinction in one of the most inbred bird species in the world, the Chatham Island black robin. We investigated whether long-term isolation and severe inbreeding led to changes in mutational load, and also examined the genomic consequences of the translocation event of 1979–1990. Overall, we found evidence for a significant decrease in genome-wide heterozygosity, as well as increase in inbreeding and realised mutational load, consistent with the reported bottleneck to a single breeding pair [41]. We also found partial evidence for a temporal reduction in total mutational load for highly deleterious mutations when comparing the historical samples with those of the two surviving populations. Furthermore, the translocation induced only small changes in the frequency of deleterious alleles.

### Population structure and past demography

Population structure analyses revealed large heterogeneity in the historical population, with two main genetic clusters. Incomplete and erroneous sampling location information, a common issue in museomics (e.g., [42, 43]), can lead to inconsistencies between sample labelling and clustering and make the resolution of past population structure challenging. However, historical samples were grouped into either a pre-1894 or post-1894 cluster (to the exception of BRH13 collected in 1871, grouping with post-1894 samples). Black robins were extirpated from Rangiaotea/Pitt Island well before 1863 and from Maung’ Rē by 1894 [29, 44]. Therefore, we cannot exclude the possibility that samples collected prior to 1894 originated from Maung’ Rē and those collected after originated from Tapuaenuku/Little Mangere. The two modern populations formed a single cluster that was highly homogeneous and distinct from the historical population, which is consistent with strong genetic drift caused by the severe bottleneck comprising a single breeding pair [28–31].

Overall, our PSMC analysis indicated that the black robin experienced a continuous population decline, most likely associated with past warming periods and global sea level rise [45]. The onset of this decline seems to have coincided with the Kaihinu warming period around 100–90 ka BP, and continued well after the LGM (i.e., 25–18 ka BP) and into the mid-Holocene [46–48]. We hypothesise that a decrease in habitat availability and connectivity among islands of the archipelago, as suggested for Chatham Islands skink (*Oligosoma nigriplantare nigriplantare*; [49]) could have induced the observed signature of population decline [47]. The curves corresponding to historical birds in the PSMC also indicated an increase in N_e_, possibly caused by increased gene flow or hybridisation between divergent lineages (see [50]) ~ 30 ka BP, which is close to the time period of LGM sea-level minimum when the islands formed a single landmass [47, 48]. It is however unclear why the modern genomes did not retain the same signature of demographic increase observed in the historical genomes. While inbreeding should not bias PSMC curves [51], we cannot dismiss recent and extreme inbreeding as factors obscuring some of the history of black robin. Alternatively, owing to possibly erroneous sampling information from museum specimens, historical genomes may originate from populations with different population histories than the surviving populations.

The past demography inferred using the SMC + + also indicated a continuous decline, most apparent in historical genomes, but no population decline over the past few centuries in either modern or historical genomes. In contrast, the most recent demographic history inferred with GONE showed a decline dating back ~ 150–300 years BP, consistent with the severe decline starting in the mid- to late- 1800s [28]. However, neither SMC + + nor GONE detected a decline coinciding with Moriori settlement of the Chatham Islands c. 1450 CE [52], which suggests that the first introduction of Polynesian rats (kiore, *Rattus exulans*) had a limited impact on black robin populations [28]. This is consistent with previous genetic and observational studies [44, 53–56] that indicate that the establishment of kiore was only restricted to some parts of the archipelago. For instance, even though Moriori settled on Rangiaotea/Pitt Island at various times, there is no indication that kiore established there before European arrival [53]. Consequently, it is likely that black robin survived through centuries of Moriori contact including on Tapuaenuku/Little Mangere and even on the larger and inhabited Rangiaotea/Pitt Island well into the 19^th^ Century, until habitat modification and the introduction of cats by Europeans in the early 1800s induced a severe population decline [28].

### Genome-wide diversity, inbreeding and mutational load

The near-extinction event in the early 1980s is clearly reflected in several genomic parameters of the modern populations, as the amount of genome-wide diversity lost and the magnitude of increased inbreeding are greater or similar compared to several other species of conservation concern, including the kākāpō, another critically endangered bird from New Zealand with a similar population history to black robin [12]. It is worth noting that the longest ROH observed in the modern black robins (i.e., 43.6 Mb) also corresponds to the longest scaffold in our assembly. Therefore, ROH lengths may even be greater and possibly extend to complete chromosome lengths. We found only limited evidence for a reduction of total load in the black robin, which suggests that purifying selection or drift have not induced drastic changes in load since the 1800s. While there was some evidence for a reduction in the ratio of Moderate impact variants (i.e., R_xy_), but not total count, our data also indicated that mutations in conserved regions for low to intermediate GERP score (i.e., GERP < 3, ~ 81% of all positive GERP scores) sites had been reduced in modern birds. This is somewhat unexpected as selection against moderately and weakly deleterious variation should be less efficient than against highly deleterious variation [10].

Theory, genome-informed simulations, and empirical data on other severely bottlenecked populations indicate that purging is more likely to take place for highly deleterious variation, and that severe population declines are likely to lead to an accumulation of additive genetic load for moderately and weakly deleterious variation [9–17]. However, in cases of gradual bottlenecks, or with a history of long-term small population size, rare recessive deleterious variation including mildly deleterious variants can also be purged, due to more frequent exposure in homozygous state compared to larger populations, as shown in kākāpō [12]. Such a scenario is possible for the black robin. Indeed, a founder effect associated with the colonisation of the Chatham Islands by the ancestor of black robin 1–4 million years (ma) BP [57, 58] may have resulted in high inbreeding and facilitated purging of ancestral load. Furthermore, because of the limited dispersal capabilities of black robin across open areas [29], its populations were likely fragmented and isolated on several islands for thousands of generations as sea level rose during the Holocene [47], similar to Chatham Islands skink [49]. Black robin populations, including the last one remaining on Tapuaenuku/Little Mangere [28], may thus have had small N_e_ for prolonged periods of time and hence been exposed to recurrent purging of load [12], which could have made the black robin more resilient to subsequent bottlenecks. Interestingly, while rare, we found some large ROH (max ROH length ~ 31 Mb) in the historical black robin genomes, which implies that inbreeding of close relatives occurred already prior to the 1900s bottleneck. These inbreeding events may have catalysed earlier periods of purging associated with the local introduction of kiore by Moriori c. 1450 CE, or by the introduction of cats or through habitat degradation by Māori or Europeans [28]. If this hypothesis is true, it would explain the limited temporal decline in load observed since the 1800s. It is even possible that while most deleterious alleles may have been purged in the past, those that remain may only have a limited impact on fitness and are thus retained and tolerated in the population (e.g., [59]), but see [32].

The translocated population on Hokorereoro is the only modern population showing a significant, yet small, reduction in total load for High impact variants in coding regions, while Maung’ Rē showed a small but non-significant reduction compared to the historical genomes. Moreover, the population on Hokorereoro has a lower realised load compared to the source population on Maung’ Rē, reflecting its lower inbreeding. Whether this overall reduction of load is due to purifying selection facilitated by high exposure of deleterious variation in homozygous state or due to a founder effect during translocation, remains unclear. These results indicate that the establishment of the Hokorereoro ‘insurance population’ may not have worsened the potential negative effects of load. However, phenotype data are essential to determine the magnitude of inbreeding depression in the population on Hokorereoro relative to the population on Maung’ Rē [35]. While there were observable changes in frequencies of some High impact alleles between the modern populations, these differences were rather minimal. The two populations shared a majority of High impact variants (i.e., ~ 90% of total and ~ 50% of fixed), possibly because these were already fixed in Maung’ Rē before the translocation to Hokorereoro. Moreover, there were deleterious variants showing either a lower or higher frequency in Hokorereoro compared to Maung’ Rē, suggesting that the translocation simultaneously had potential positive and negative effects on the fitness of the population. This indicates that the establishment of a third ‘insurance population’ could also represent a risk of an increase in load and suggests that individuals with the lowest total load should be prioritised for future translocations. It is also worth noting that since deleterious variants fixed in both populations cannot be lost via genome-guided translocations, using genetic engineering to introduce lost, putatively adaptive or beneficial variants (e.g., identified through GWAS) could possibly serve as a means of increasing population viability [60]. For instance, sperm transfection assisted gene editing (STAGE), which combines sperm injection with gene editing technologies such as CRISPR, has been successfully applied in chicken and has been proposed as a more straightforward approach for non-model organisms, including species of conservation concern in need of genetic rescue [61].

Although we did not observe a substantial temporal change in mutational load, two previous studies based on a pedigree spanning 20 years support inbreeding depression in black robin and showed a negative relationship between inbreeding (both juvenile and parental) and juvenile survival, suggesting fixation of deleterious alleles [33, 35]. However, paradoxically, Weiser et al. [33] also found that fledging survival was improved for chicks with highly inbred mothers who were closely related to the father, compared to when the father was more distantly related to the mother [33]. It was hypothesised that inbreeding in black robins is so high that offspring inherit multilocus, ‘proven genotypes’, that are identical or very similar to one of their parents due to long stretches of shared ancestry between the parents [33]. Although the pedigree study assumed that extra-pair paternity (EPP) was low in black robin [33], a recent study estimated an EPP rate of 20% [62]. Combining pedigree with phased haplotype data could thus allow to test whether EPP impacts the inheritance pattern of such ‘proven genotypes’ and can reduce individual fitness. Overall, the results of these two studies [33, 35] as well as ours further underlines the complex dynamics of mutational load. While purging of highly deleterious variation can occur [10, 11], small populations can still experience inbreeding depression through the accumulation of mildly deleterious mutations underpinning polygenic complex traits (e.g., longevity, survival and reproduction, behaviour [32]).

Using genetic rescue as a means of preventing population extirpation has proven successful on several occasions (e.g., [26, 63–68]). Even translocations between inbred populations can increase fitness of recipient populations as recessive deleterious alleles in one population become masked by alleles from the second population [69]. While there is a potential risk of introducing deleterious variation from a large outbred into a smaller inbred population [70], this risk should be limited in black robin since both populations have comparable mutational load. Moreover, because the extant populations show different allele frequencies for some High impact variants, individuals with a lower load could be prioritised for translocations and contribute, to some extent, to genetic rescue via reciprocal translocations [34]. Although dispersal between the modern populations separated only by a ~ 300 m. water gap has never been observed [29], we cannot exclude future dispersal as habitat is restored, meaning that genetic rescue may also occur naturally in the future. Alternatively, since no other conspecific populations exist [28, 29], genetic rescue of black robin through hybridisation with a closely related species could also be considered. While this approach is contentious, it has proven successful at the subspecies level for the highly inbred Florida panther [65, 71]. The same approach has been proposed across species boundaries, by crossing the critically endangered orange-bellied parrot (*Neophema chrysogaster*) with one of its sister species, from which it diverged between 2.4 and 3.5 ma BP [72]. Interspecific hybridisation occurred as an unintended consequence of allowing Chatham Island tomtit foster-parents to raise black robin chicks full-term to fledging as a means of increasing reproductive output [28, 70]. All hybrid offspring were culled however, due to concerns of genetic swamping in black robin populations, although concerns about undetected introgression remained [28]. A subsequent study found no tomtit introgression in black robin, suggesting that either all hybrid offspring were indeed culled or that hybrids that were not culled were not reproductively successful, owing to selection against hybrids [70]. Another alternative candidate for genetic rescue of black robin would be the New Zealand robin (*P. australis*), which may have diverged from black robin after the emergence of Chatham Islands 1–4 ma BP [57] but seems to share more morphological and behavioural affinities with black robin compared to the Chatham Island tomtit [28]. Yet, it is important to stress that such a long divergence time may preclude interbreeding between these two species. Nevertheless, whenever considering genetic rescue, it is essential to consider the risk for negative effects such as outbreeding depression or genetic swamping associated with interspecific hybridization [73, 74]. Therefore, such an intervention must be based on a number of important biological evaluations related to the feasibility of a potential crossing, including ecological and behavioural similarities as well as using phylogenetics to identify the most closely related species [72].

## Conclusion

In this study, we analysed 52 Chatham Island black robin genomes, including 17 pre-bottleneck genomes from the 1900s to investigate the genomic consequences of a near-extinction event. We found a substantial loss of genome-wide diversity and increased inbreeding, but only partial evidence for a decrease in mutational load. We hypothesise that the only surviving population, which declined to five birds, including only a single pair producing viable young between 1979 and 1981, might have undergone purging of deleterious variation already prior to this bottleneck. This may have been facilitated by ancestral population fragmentation and isolation on various small islands of the archipelago for 1000s of generations. Our data also suggest that the establishment of a second population on Hokorereoro did not cause any substantial changes in the frequency of deleterious variants. However, even if ancestral purging events occurred, most of the remaining detrimental variation is shared between populations. To minimise the risk of further fixation of detrimental variation and maximise the chances of the species’ recovery, individuals with the lowest load should be selected for future translocations or genetic rescue attempts. Moreover, while dispersal has not been observed between the two modern populations, tracking the genomic consequences of potential dispersal events would be essential to monitor the evolution of this load in the future.

## Methods

### Sampling, DNA extraction, library preparation and sequencing

We obtained 35 blood samples from the extant populations (i.e., the modern populations) on Mangere/Maung’ Rē and South East Island/Rangatira/Hokorereoro Islands as well as 17 toepad samples from historical individuals from the Chatham Islands (Table S1). Note that throughout the manuscript, we only refer to Moriori island names for simplicity. DNA from blood samples was extracted using a Kingfisher robot (Thermo Fisher Scientific) and following the Kingfisher blood & tissue extraction protocol according to the manufacturer’s instructions. DNA from toepads was extracted using a DNeasy Blood & Tissue Kit (Qiagen, Hilden, Germany).

Library preparation from modern DNA extracts was performed using a PCR-free protocol at the Science for Life Laboratories (SciLifeLab), Stockholm. We prepared libraries for historical samples as described in Dussex et al. [75], following [76] and including USER treatment to remove deaminated bases [77] as well as 5–6 independent PCRs to maximise complexity. Libraries were sequenced on a NovaSeq S4 using a 2 × 150 bp and 2 × 100 bp setup for modern and historical libraries, respectively.

### De-novo genome assembly

We extracted DNA from a blood sample of a female black robin *P. traversi* (sample ID: 09_131; band ID: 98,938; Rangatira 2014). The extracted material was sent to the Swedish National Genomics Infrastructure (NBIS), Stockholm, to be prepared as a linked-read library using a Chromium genome v2 kit from 10X Genomics. The resulting library was sequenced on a S4 flowcell on the Illumina NovaSeq 6000 sequencer, where it was pooled to occupy approximately 25% of the capacity of one lane. The sequencing run yielded 1255.42 M passing clusters (i.e., read-pairs), or ~ 351X sequencing depth.

The sequenced data was assembled in Supernova v2.1.1 [78], using parameters “–maxreads = all” and “–accept-extreme-coverage”. Assembly evaluation was performed using QUAST v4.5.4 [78, 79] and BUSCO v5.0.0 [80] using the aves_odb10 ortholog dataset.

To identify Z- and W-linked scaffolds in the black robin assembly, we used the snakemake workflow findZX (https://github.com/hsigeman/findZX/; [81]) and processed whole genome data from three male and three female black robin. Briefly, this approach identifies sex-linked genome regions across reference genomes by scanning for regions that differ between sexes in terms of genome coverage and/or heterozygosity (see Supplementary information; [81]. We used the output from the pipeline to group scaffolds into four categories based on female-to-male coverage and heterozygosity differences as well as synteny information to the zebra finch genome: (1) autosomal, (2) Z-linked and (3) W-linked or (4) unsure (could not be assigned to category 1–3; Fig.S1-2, Table S2). Finally, we identified repeats using repeatmodeler v1.0.11 and repeatmasker v4.0.7 [Smit, A.F.A. and Hubley, R. (2008–2015) RepeatModeler Open-1.0, http://www.repeatmasker.org/] and CpG sites using the GenErode bioinformatics pipeline [82].

### Data mapping

Mapping and variant calling for historical and modern data was done using a beta version of the GenErode bioinformatics pipeline [82]. Briefly, adapter trimming was done with trimmomatic v0.32 [83] for modern samples while we used Seqprep v1.1 (https://github.com/jstjohn/SeqPrep) to trim and merge forward and reverse reads for historical samples. Next, reads for modern and historical data were mapped using BWA v0.7.17 mem and aln algorithms respectively, while sorting and removing PCR duplicates were done using SAMtools v1.12 [84]. Finally, reads were realigned around indels using GATK IndelRealigner v3.4.0 [85]. We then estimated depth of coverage using SAMtools depth, as well as filtering out bases with quality (-Q) and reads with mapping quality (-q) < 30.

Variant calling was done with bcftools mpileup command and bcftools v1.8 [84, 86]. We used a minimum depth of coverage (DP4) of ~  $\frac{1}{3}$ of the average depth of coverage, and filtered SNPs by base quality QV ≥ 30 and those within 5 bp of indels. SNPs in heterozygous state were filtered out if the allelic frequency fell outside an allelic balance (i.e., number of reads displaying the reference allele/depth) of < 0.2 and > 0.8 in order to avoid biases caused by contamination, mapping or sequencing error. After merging all individual vcf files, we masked CpG and repeat sites, and excluded scaffolds linked to Z and W chromosomes with the bcftools view command.

We obtained a total of 2,731,301 high-quality SNPs across our 52 genomes, hereafter referred to as the '*complete*’ dataset that was used as input for population structure analyses. For all other analyses involving population comparisons including demographic analysis (i.e., SMC + + , GONE), we used PLINK v1.9 [87] to retain only SNPs called in the 42 individuals from all populations (i.e., historical, *n* = 13; Maung’ Rē, *n* = 9; and Hokorereoro, *n* = 20), hereafter referred to as the ‘*three populations*’ dataset, with a genome coverage $\ge$ 11X, making for a total of 535,269 SNPs.

For analyses that required the calling of variants relative to an ancestral allele (e.g., SMC + + , GERP scores estimation, SnpEff), we replaced the reference allele in the vcf file with that of Southern scrub robin (*Drymodes brunneopygia*; GCA_013400955.1) as described in [12] to avoid a bias associated with modern genomes being more identical to the genome assembly than historical ones (Fig. S3). After filtering the variants set for missing data, we obtained a total of 229,222 SNPs in the 42 individual genomes.

### Data analyses

#### Population structure

Using the ‘*complete*’ dataset, we first performed a PCA analysis in PLINK v1.9. Secondly, we used ADMIXTURE v1.3.0 [88] to estimate individual-based ancestry and identify genetic clusters (K = 1–5). This approach assumes that individuals are unrelated and uses a cross-validation procedure to determine the best number of possible genetic groups present in the dataset.

#### Past demography

To infer past demography of black robin we used the Pairwise Sequentially Markovian Coalescent (PSMC) v0.6.5 [89] to estimate past fluctuations in effective size (N_e_). This model identifies historical recombination events across a single diploid genome to infer the time to the most recent common ancestor (TMRCA) between genome segments. Assuming that pairwise sequence divergence is proportional to the timing of the coalescent events, parts of the genome with low heterozygosity indicate recent coalescence while regions of high heterozygosity correspond to more ancient coalescent events. Similarly, because the rate of coalescence is inversely proportional to N_e_ [89], it can then be used to estimate temporal changes in N_e_.

We generated consensus sequences for all autosomes of one high-coverage genome per population (BRH01, BRH11, BRH12, BRM03, BR75) using the SAMtools mpileup [84] command with base and mapping quality filters (-Q 30, -q 30) and the vcf2fq command from vcfutils.pl. We excluded sites with depth < 5X. We used a strict threshold of maximum depth excluding positions with more than two times the average coverage estimated for each genome. We set N (the number of iterations) = 25, t (Tmax) = 15 and p (atomic time interval) = 64 (4 + 25*2 + 4 + 6, for each of which parameters are estimated with 28 free interval parameters) for the inference of TMRCA between each chromosome from each individual genome. We scaled population parameters assuming a generation time of 2 years [28] and a substitution rate of 2.3×10^-9^ substitutions/site/generation based on a direct estimate for flycatcher (*Ficedula albicollis*) [90].

Because the PSMC relies on a single diploid genome, there are only a few coalescent events in the recent past, thereby limiting the power in detecting recent past population fluctuations (i.e., < 10 ka BP, [89]. To circumvent this limitation, we used two different approaches to infer recent fluctuations in N_e_. First, we used the SMC + + v.1.15.2 [91] which also uses sequential Markov coalescent (SMC) simulations, but relies on unphased genome data from multiple genomes instead of relying on one single diploid genome, thereby increasing the number of recent coalescent events. Using the same substitution rate and generation time as for the PSMC, past demography was estimated using the ‘estimate’ command with –em-iterations 5000 and –thinning 1300. Second, we used GONE [92] which estimates changes in N_e_ from the observed spectrum of linkage disequilibrium (LD; https://github.com/esrud/GONE). We only retained the 85 largest autosomes and used the following parameters: PHASE = 2; cMMb = 1; DIST = 1; NGEN = 2000; NBIN = 400; MAF = 0.0; ZERO = 1; maxNCHROM = 85; maxNSP = 50,000; hc = 0.05; REPS = 40; threads = -99. For this analysis, we grouped Maung’ Rē and Hokorereoro into a single modern population (*n* = 29).

### Heterozygosity and inbreeding estimates

We estimated heterozygosity for our ‘*three populations*’ datasets. We first used mlRho v2.7 [93] to estimate the individual mutation rate (θ), which can then be used as an approximation of genome-wide heterozygosity (measured here as the number of heterozygous sites per 1,000 bp) under the infinite sites model. We filtered out bases and reads using -Q 30 and -q 30 filters respectively and positions with root-mean-square mapping quality (MQ) < 30 from the historical and modern bam files with SAMtools mpileup. We also filtered out sites with depth < $\frac{1}{3}$ (i.e., 5X) of the average depth of coverage and higher than 2X the average coverage across all our genomes to avoid calling false heterozygous sites due to structural variation and erroneous mapping. Nucleotide diversity ($\pi$) was estimated using a sliding window of 10 kb with vcftools v.0.1.15 [94] for the 50 largest scaffolds ($\ge$ 50 Mb).

We identified Runs of Homozygosity (ROH) regions across the genomes and calculated individual inbreeding coefficients (F_ROH_) using the sliding-window approach implemented in PLINK v1.9. Because ROH identification is sensitive to input parameters, we ran the analysis with three different sets of parameters: a sliding window size of 100, 250 or 500 SNPs (*homozyg-window-snp 100/250/500*); no more than 1, 3 or 5 heterozygous site per window to assume a window as homozygous (*homozyg-window-het 1/3/5*); at least 5% of all windows including a given SNP to define the SNP as being in a homozygous segment (*homozyg-window-threshold 0.05*); a homozygous segment was defined as a ROH if the segment included ≥ 25 SNPs (*homozyg-snp 25*) and covered ≥ 100 kb (*homozyg-kb* 100); the minimum SNP density was one SNP per 50 kb (*homozyg-density 50*); and the maximum distance between two neighbouring SNPs was ≤ 1,000 kb (*homozyg-gap 1,000*). Finally, we set the value at 750 heterozygous sites within ROH (*homozyg-het 750*) in order to prevent sequencing errors to cut ROH. The inbreeding coefficient F_ROH_ was then estimated as the overall proportion of the genome (autosomes only) contained within ROH. The main results are based on: *homozyg-window-het* 5, *homozyg-window-snp* 500 (see Supplementary Information).

We statistically compared heterozygosity and F_ROH_ between populations using Tukey HSD tests in R [95].

### Estimation of mutational load using conservation scores

Since genetic load cannot be estimated in the absence of fitness data, we estimated relative mutational load as a proxy for genetic load in each individual genome from the ‘*three populations*’ dataset. First, we estimated the number of derived alleles at sites that are under strict evolutionary constraints using genomic evolutionary rate profiling scores (GERP) with the GERP + + software [96], as described in von Seth et al. [40] and Dussex et al. [12]. Briefly, GERP + + identifies evolutionary constrained elements in multiple alignments of a user-defined dataset by quantifying the amount of substitution deficits (e.g., substitutions expected to have occurred if the element were neutral DNA, but did not occur because the element has been under functional constraint) by accounting for phylogenetic divergence. Thus, sites with few substitutions across the phylogenetic tree of the user-defined dataset will be allocated higher GERP scores, and represent highly conserved regions, whereas sites with numerous substitutions across the data set will be allocated lower GERP scores and are putatively neutral. We included heterozygous (counted as one allele) and homozygous positions (counted as two alleles) even though the mutational effect of heterozygous positions also depends on additional assumptions about dominance coefficients.

To identify highly conserved regions in the black robin, we aligned and reconstructed the phylogeny of 124 avian genomes including black robin (Table S3) from GenBank by using TimeTree [96, 97] which estimates the divergence times based on automated literature searches. Each of these genomes were then converted into fastq-format (50 bp reads) and realigned against the black robin reference genome using BWA mem v0.7.13 [98, 99], slightly lowering mismatch and gaps penalty scores (-B 3, -O 4,4). Additionally, we filtered out all reads from the processed bam files aligning to more than one genomic location using Samtools v1.8 [84]. Next, we converted each alignment file to fasta-format using htsbox v1.0 and the following parameters: -R -q 30 -Q 30 -l 35 -s 1, https://github.com/lh3/htsbox). GERP + + was then used to calculate conservation scores (GERP scores) for each site in the genome for which at least three bird species could be accurately aligned to the black robin reference. We considered mutations at sites with a GERP score within the top 1% (i.e., > 5.88) of the GERP score distribution as highly deleterious, and mutations at sites with low GERP scores (< 1) as neutral.

Next, the distribution of GERP scores was divided into categories (i.e., 0 < 1, 1 < 3, 3 < 5, > 5, and > 5.88), and for each category and individual, the following formula was used to calculate relative mutational load: (sum of GERP scores of derived alleles, multiplied by 2 at homozygous sites) / (sum of derived alleles, homozygous sites counted as 2). Higher relative mutational load values indicate that a relatively larger proportion of derived alleles is found at conserved genomic sites, thus indicating higher mutational load. We statistically compared the relative mutational load based on GERP-scores for the ‘*three populations*’ dataset using a Tukey HSD test in R [95].

### Estimation of mutational load in coding regions

We used SnpEff v4.3 [100] to annotate synonymous and non-synonymous nucleotide substitutions in coding regions for black robin. We annotated the Chatham Island Black robin genome using SPALN2 as described in Lord et al. [101]. Briefly, we extracted 15,342 gene models by collapsing a reference protein set for zebra finch (*Taeniopygia guttata*; GenBank: GCA_000151805.2) following Uniprot90 guidelines using a custom script. We only retained sequences with at least 90% sequence identity to, and 80% overlap with, the longest sequence. Next, we used the exon-aware, protein-to-genome aligner SPALN2 [102] and the zebra finch proteome to predict black robin proteins to generate 12,771 gene models. After removing gene models with in-frame STOP codons using the -V option of Cufflinks v2.2.1 [103], we obtained a total of 10,720 genes. Functional annotation of these gene models was done with eggNOGmapper v4.5.1, a method that uses fast orthology assignments upon precomputed protein clusters within a phylogenetic context [104]. We selected ‘*Aves*’ as taxonomic scope, the ‘Restrict to one-to-one’ and the ‘Use experimental terms only’ options to prioritise precision, quality of matches and also to remove multiple matches which could represent pseudogenes. Overall, we obtained 9,891 annotated gene models.

We then generated a database for *P. traversi* using the protein sequences extracted from our annotation. We first identified putative deleterious variants by allocating them to three different impact categories as defined in the SnpEff manual: a) Low: mostly harmless or unlikely to change protein behaviour (i.e., synonymous); b) Moderate: non-disruptive variants that might change protein effectiveness (i.e., missense); c) High: variants assumed to have high (disruptive) impact on protein, probably causing protein truncation, loss of function or triggering nonsense mediated decay and including stop gained codons, splice donor variant and splice acceptor, start codon lost [100]. Because we only used sites covered in all individuals, we counted the number of variants in these three categories separated by homozygous and heterozygous state and did not need to use bootstrapping of allele counts. We then estimated individual mutational load for Moderate and High impact variants in two ways. First, we calculated the individual total load as the sum of variants for each category as described in Dussex et al*.* [12]. Secondly, we calculated the individual realised load (i.e., total number of homozygous variants of category *i* divided by twice the total number of segregating sites for category *i*) as described in Mathur & DeWoody [14]. By taking into account the mode of dominance, this estimate allows us to estimate the proportion of total load that is realised (i.e., expressed) in each individual. We compared the differences in load among the ‘*three populations*’ using Tukey HSD in R [95]**.**

We then estimated the difference in frequency of Moderate and High impact variants for each population pair comparison by calculating the R_xy_ ratio as described in Dussex et al*.* [12]. We calculated for each site i of the observed allele frequency in Population x as f^x^_i_ = d^x^_i_ /n^x^_i_, where n^x^_i_ corresponds to the total number of alleles scored in population x and d^x^_i_ to the total number of derived alleles. Similarly, we define f^y^_i_ for population y. For each C category of variants we then calculated:

$${Freq}_{pop-x}\left(C\right)=\sum_{i \in C}f\frac{x}{i}(1-{\mathrm{f}}_{i}^{y})$$

We then calculated the R_xy_ = Freq_pop-x_ / Freq_pop-y_ ratio. R_xy_ equal to 1 corresponds to no change in frequency between two populations, whereas R_xy_ < 1 or > 1 corresponds to a decrease or an increase in frequency in population x relative to population y, respectively. The variance in the R_xy_ ratio was estimated with a jack-knife procedure across chromosomes in R. We only included sites where at least one out of all alleles is derived in both populations in the estimation of R_xy_.

Finally, we ran the SnpEff analysis to identify High impact variants in coding regions for a reduced ‘*two populations’* (modern birds only; *n* = 29) dataset. Since our datasets were filtered for missing data, performing this analysis for the ‘*two populations*’ dataset only, allowed us to maximise the number of variants discovered and thus provide a more comprehensive understanding of gene and biological functions affected by mutational load. After excluding Z- and W-linked scaffolds (Table S2) we obtained 1,325,267 SNPs. We then estimated the frequency of each High and Moderate impact allele per population using PLINK v1.9.

### Gene ontology

We performed a functional classification and overrepresentation test for genes containing High variants identified with the SnpEff using a Gene Ontology analysis implemented in Panther v17 [105] using *Gallus gallus* as reference set. We performed this analysis only on the 29 genomes from the ‘*two populations*’ dataset.

## Supplementary Information

**Additional file 1. **

**Additional file 2. **

## Data Availability

This Whole Genome Shotgun project has been deposited at DDBJ/ENA/GenBank under the accession number JAHLSL000000000. The version described in this paper is version JAHLSL010000000 (GenBank assembly accession: GCA_025920805.1; BioProject: PRJNA735117). Resequencing data has been deposited on the European Nucleotide Archive (ENA: ﻿https://www.ebi.ac.uk/ena; Proj. number: PRJEB46470).
