# Supplementary material for: Genomic trajectories of a near-extinction event in the Chatham Island black robin
Source: BMC Genomics. 2022 Nov 10;23:747. doi: 10.1186/s12864-022-08963-1 (PMC9647977; doi:10.1186/s12864-022-08963-1)
Supplement: Supplementary file 1 — Additional file 1. [file 12864_2022_8963_MOESM1_ESM.docx]

**Supplementary Material for:**

**Genomic trajectories of a near-extinction event in the Chatham Island black robin**

von Seth et al.

1. **Sex-linked chromosome identification**

To identify Z- and W-linked scaffolds we used the snakemake workflow findZX (https://github.com/hsigeman/findZX/; accessed July 2021) to process WGS data from 3 male and 3 female black robin samples (i.e., females: BRM03, BR75, BR07. males: BR17, BR35, BRM08). The purpose of this workflow is to identify sex-linked genome regions across reference genomes by scanning for regions that differ between sexes in terms of genome coverage and/or heterozygosity. Briefly, this pipeline includes the following steps: all samples were trimmed using Trimmomatic v0.39 [[1]](https://paperpile.com/c/EDHKv8/1NXwy), then aligned to the black robin reference genome using BWA mem v0.7.17, [[2, 3]](https://paperpile.com/c/EDHKv8/hLZgd+553Zg) sorted with SAMTOOLS v1.12 [[4]](https://paperpile.com/c/EDHKv8/v7HHR) and duplicate reads were removed using Picard v2.22.1 (http://broadinstitute.github.io/picard/). Genome coverage was calculated across 5kb genome windows with bedtools multicov v2.27.1 [[5]](https://paperpile.com/c/EDHKv8/cDwdc) (only considering reads with quality scores over 20). Variants were called using Freebayes v1.1.0 [[6]](https://paperpile.com/c/EDHKv8/raZU), and low-quality variants were removed with vcftools v0.1.15 [[7]](https://paperpile.com/c/EDHKv8/UjH8Q). The number of heterozygous sites per 5kb window and sample was calculated using a custom script. Mean values for each 5kb window was calculated per sex, for both the genome coverage and heterozygosity values. Based on a synteny-analysis using the program last v1238 [[8]](https://paperpile.com/c/EDHKv8/gJfu) between the black robin reference genome and the zebra finch genome (Taeniopygia_guttata-3.2.4; GenBank assembly accession: GCA_000151805.2), the sex-specific genome coverage and heterozygosity values were translated to chromosome coordinates along the zebra finch genome. Mean (and standard deviation) values across 100kb and 1 Mb windows were calculated and plotted (Figure S1). From this analysis, we could see that the entire chromosome Z (ancestral bird sex chromosome), as well as parts of chromosome 10 (~9.3-18.3 Mb) are sex-linked in black robins. It is typical that old sex chromosome regions (such as chromosome Z, where the homologous W chromosome is almost completely gone) have very little differences in heterozygosity but very clear differences in genome coverage. Sex chromosome regions of younger age, on the other hand, often have less clear genome coverage differences but pronounced heterozygosity differences. So, these patterns are typical for a neo-sex chromosome system.

**
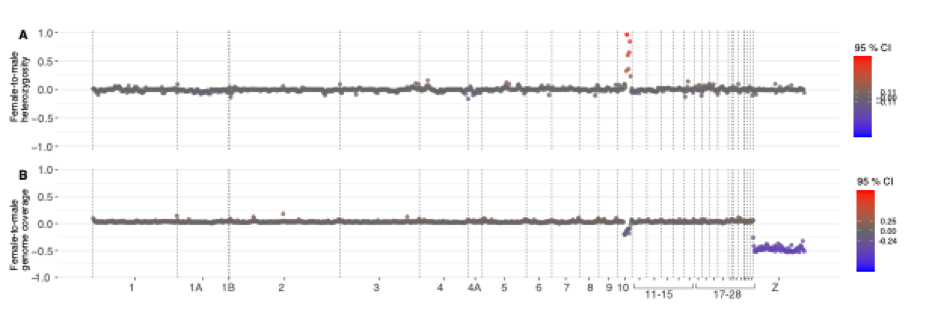
Figure S1.** Genome-wide female-to-male differences in (A) heterozygosity and (B) genome coverage values across 1 Mb windows. The entire chromosome Z, and parts of chromosome 10 are shown to be sex-linked. 95% CI were calculated based on all genome windows, and data points outside these limits are colored either red (upper outliers) or blue (lower outliers).

**
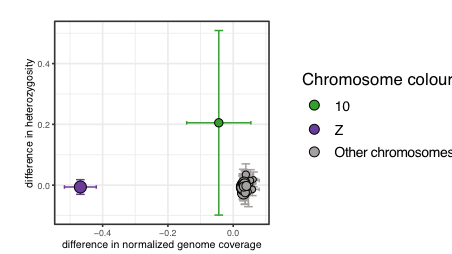
**

**Figure S2.** The data underlying this figure is the same as Figure S1, but here mean and standard deviation is calculated per chromosome (across 1 Mb windows). The figure shows that chromosome Z and chromosome 10 are the only ones with pronounced sex-differences in genome coverage and/or heterozygosity.

We then categorised scaffolds in the black robin genome into the following four groups: (1) autosomal, (2) Z-linked and (3) W-linked or (4) Unsure (could not be assigned to category 1-3). To do this, we used the genome coverage and heterozygosity values that were calculated across the 5kb windows in the black robin genome (see above). For each scaffold, we calculated per-sex median values of genome coverage (total number of reads aligned to each window from the three samples per sex) and proportion of heterozygous sites. The genome coverage values were normalised between sexes. We removed any scaffolds with upper outlier genome coverage values (>3000, threshold based on visual inspection of data), as well as scaffolds shorter than 10 kb. We assigned the scaffolds into groups based on what chromosome in the zebra finch genome they predominantly aligned to (based on the synteny-analysis; see above). Specifically, if more than 55% of a black robin scaffold aligned to one zebra finch chromosome, we assigned the scaffold to that chromosome. Scaffolds that could not be assigned to a zebra finch chromosome were labelled as “Unknown”.

We then grouped the scaffolds into the four categories (1-4, see above) based on the following criteria. (1) Autosomal scaffolds: (i) female and male coverage >1000, (ii) only scaffolds not assigned to chromosome Z or chromosome 10 (sex-linked in black robins; see Figure 1 and 2), and <5% heterozygosity difference between sexes. (2) Z-linked scaffolds: (i) Female-to-male genome coverage ratio <0.7, (ii) female and male coverage >500, (iii) only scaffolds assigned to chromosome Z or chromosome 10 or “Unknown”. (3) W-linked scaffolds: (i) female genome coverage >500, male genome coverage <100, (ii) female coverage <1500, (iii) only scaffolds assigned to chromosome Z or chromosome 10 or “Unknown”. (4) Unsure scaffold: (i) all others.

Based on this categorization, 409 scaffolds (891591097 bp) were categorised as autosomal, 106 (8622607 bp) as Z-linked, 78 as W-linked (17323089 bp) and 220 as Unsure (23564835 bp; Table S3).

**Table S3.** Number of scaffolds (and total length) belonging to each category. The total length of the Z-linked and W-linked scaffolds correspond well to what we would expect based on the neo-sex chromosome system (i.e., based on the length of chromosome Z, W, and the sex-linked part of chromosome 10).

| **Category** | **Nr scaffolds** | **Total length (bp)** | **Assigned to chr Z** | **Assigned to chr 10** | **Assigned to "Unknown"** |
| --- | --- | --- | --- | --- | --- |
| Autosome | 409 | 891591097 | 0 | 0 | 0 |
| Zlinked | 106 | 78622607 | 73 | 30 | 3 |
| Wlinked | 78 | 17323089 | 6 | 53 | 19 |
| Unsure | 220 | 23564835 | 27 | 34 | NA |

1. **Population structure, inbreeding, nucleotide diversity**


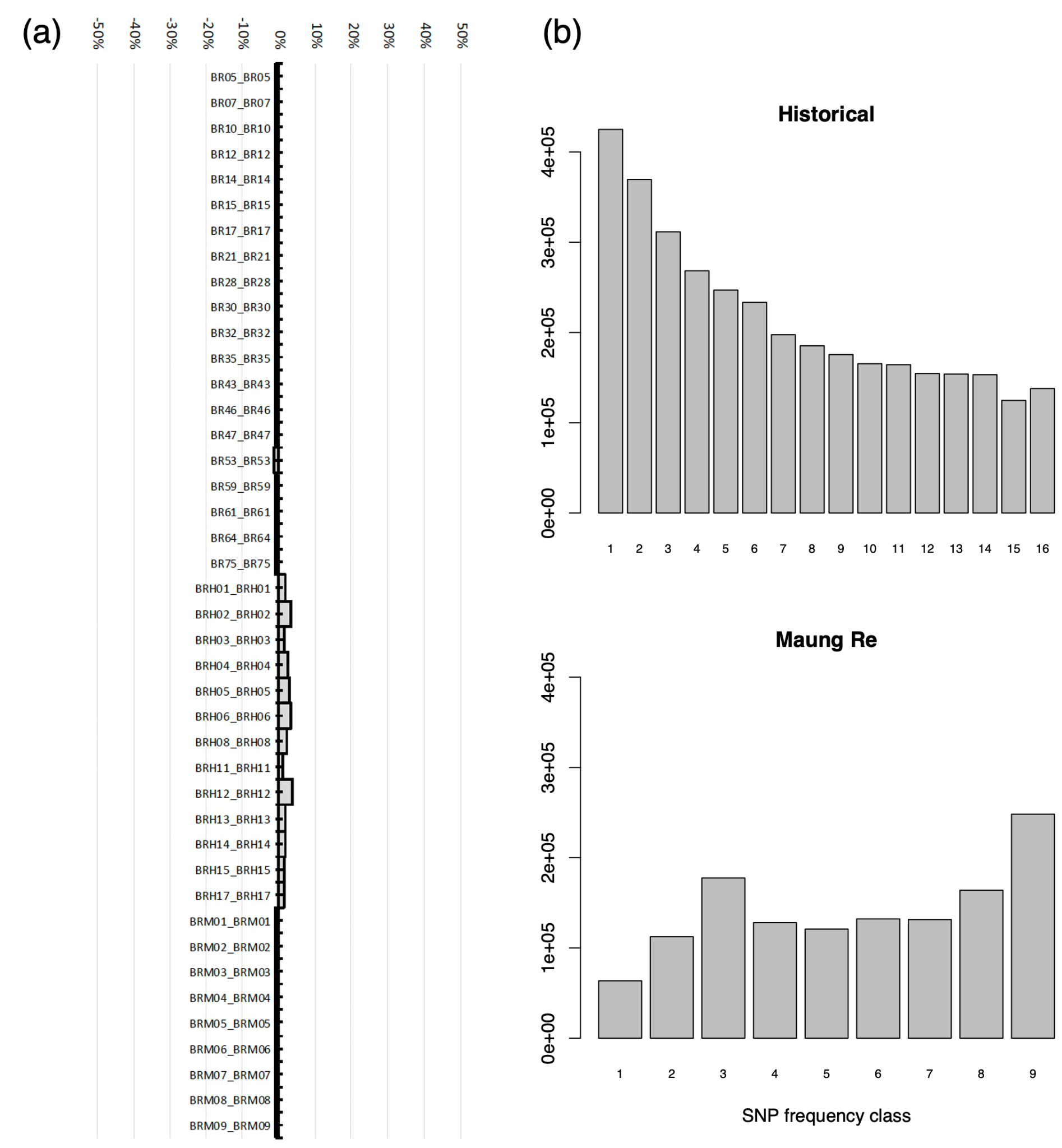


**Figure S3**. **(a)** Derived allele counts per genome as a percentage of the average after replacing the reference allele with that of *Drymodes brunneopygia* (GCA_013400955.1). **(b)** Folded Site Frequency Spectrum (SFS) for historical and modern genomes of the sole surviving population (Maung’ Rē). Less than 5% of alleles are at frequency <10% in the modern population vs 22% in the historical, reducing population level diversity by a factor of 4.6.


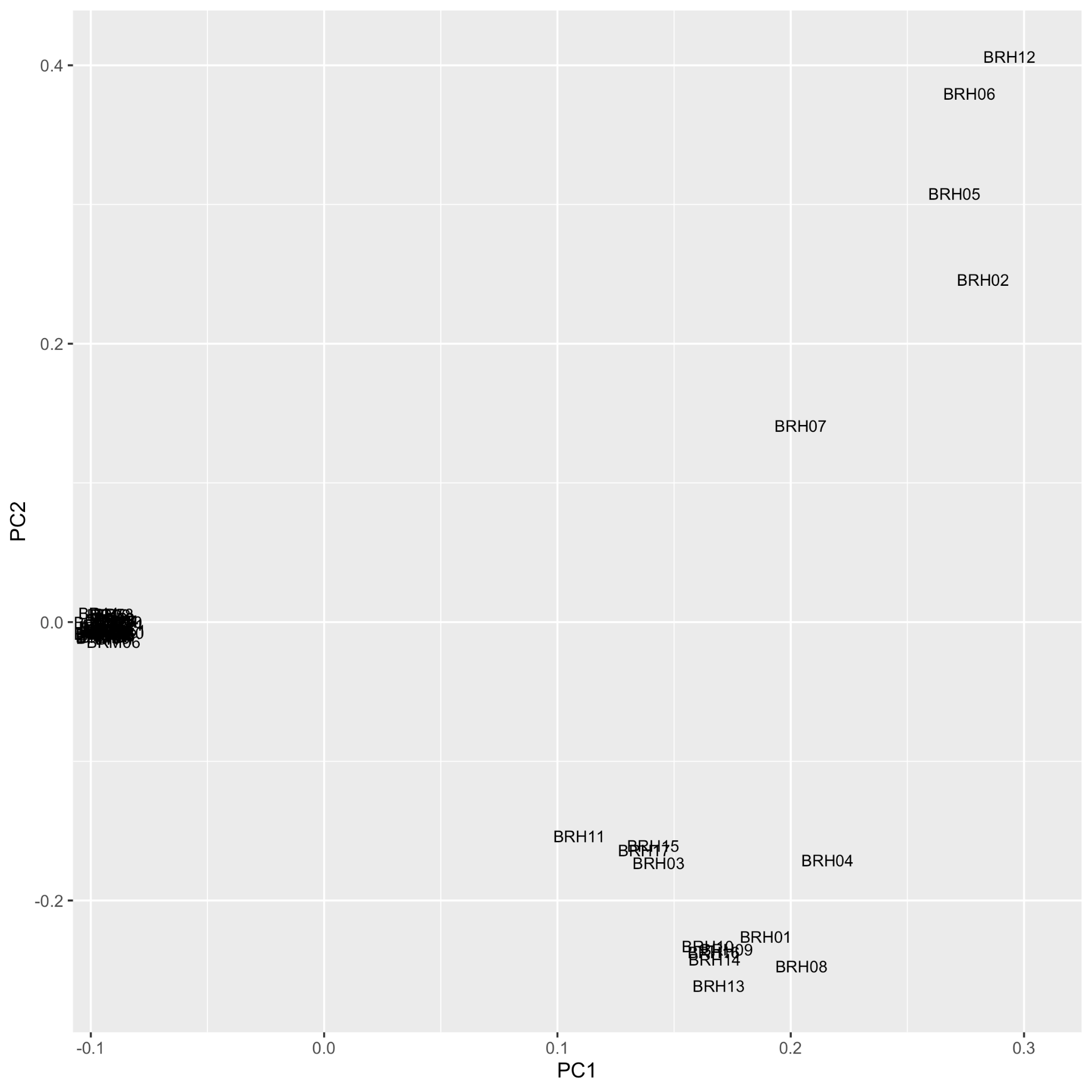


**Figure S4.** Principal Component Analysis (PCA) showing individual sample names. All modern specimens form a tight cluster at the left of the PCA plot.

**
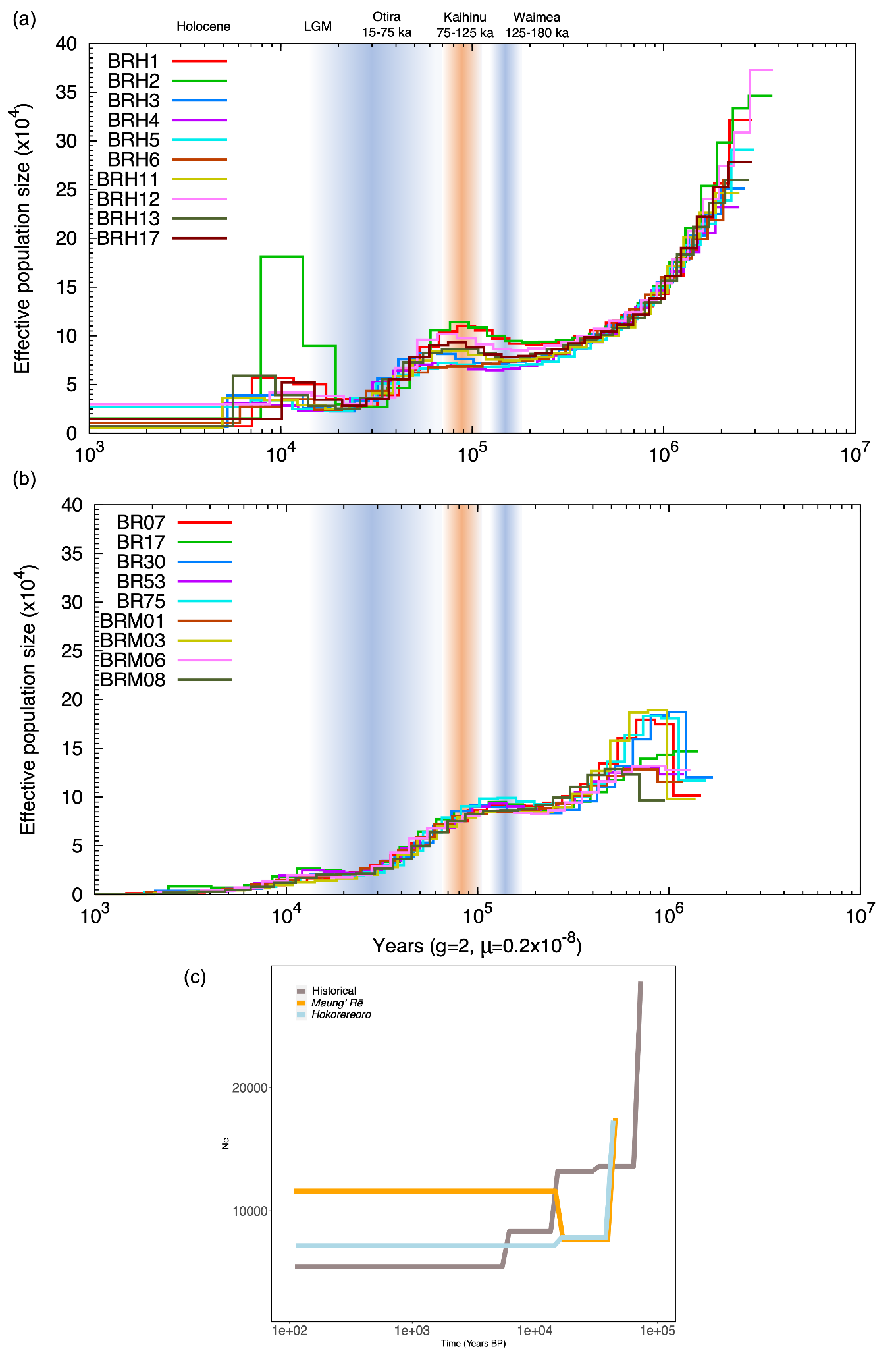
**

**Figure S5**. Demographic history of the black robin including for a subset of **(a)** historical and **(b)** modern high-coverage (≥11X) using the PSMC. Each curve represents an individual genome. Glacial and interglacial periods are shown in blue and orange, respectively. **(c)** Demographic history using the SMC++ approach. Each curve represents multiple genomes per population (Historical, n=13; Maung’ Rē, n=9; and Hokorereoro, n=20).


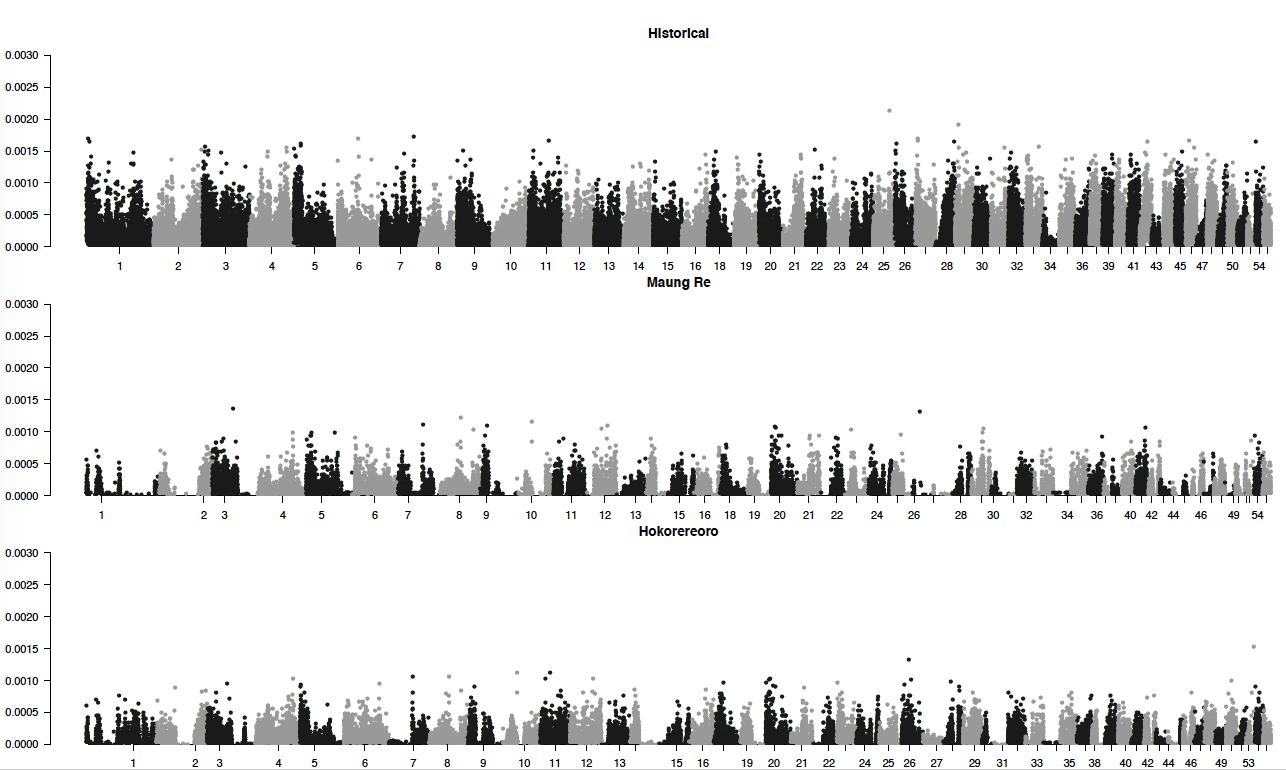


**Figure S6**. Genome-wide nucleotide diversity ($\pi$) estimated in 10kb windows for the three populations. Average Historical = 0.00023247; Maung’ Rē = 0.00013201; Hokorereoro = 0.00012614.

**
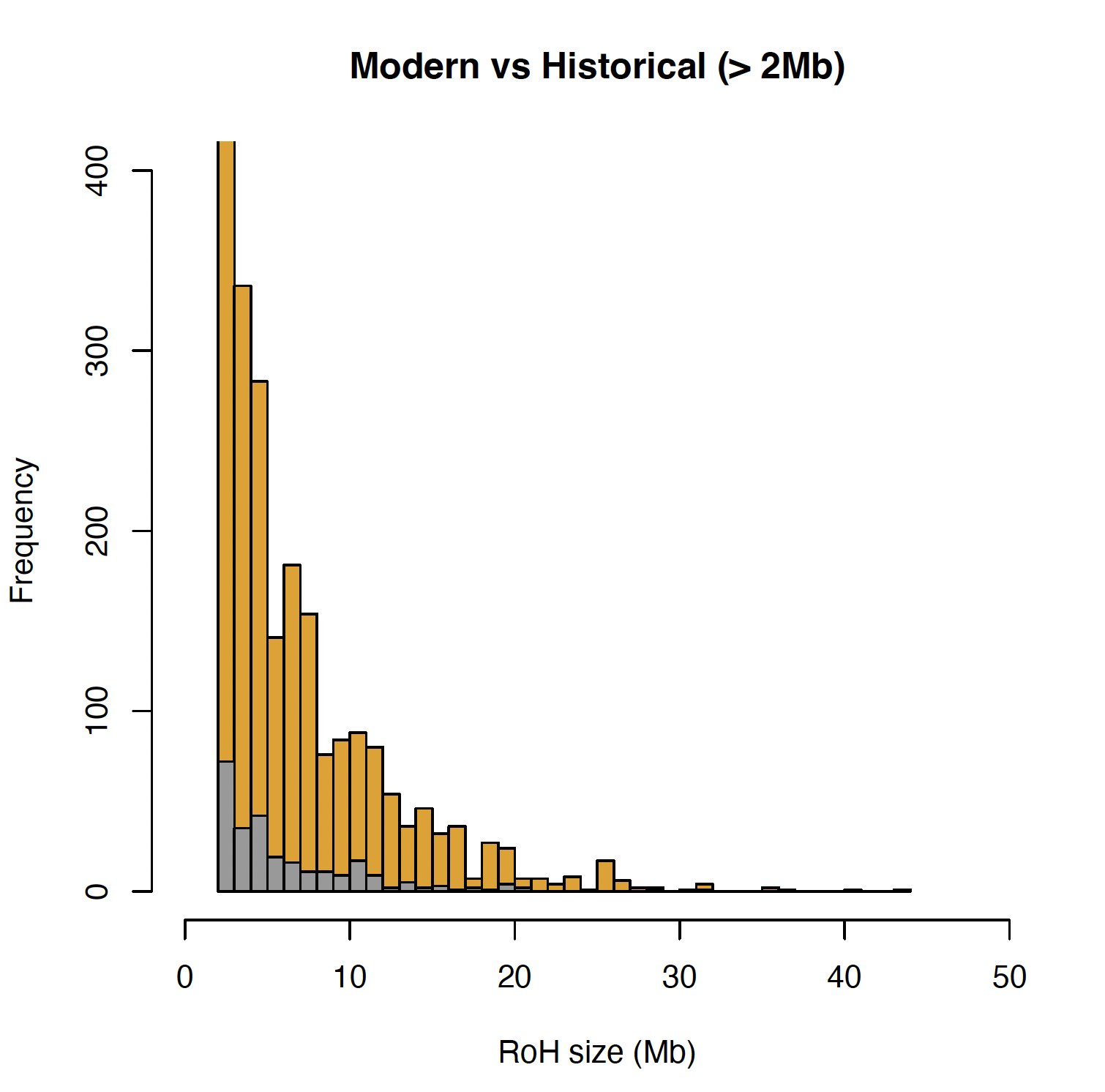
**

**Figure S7**. Frequency distribution of homozygosity (ROH) size for all ROH ≥ 2Mb for modern (yellow) and historical (grey) genomes. Max_Hist._ = 31.14; Max_Modern_ = 43.61.

(a)


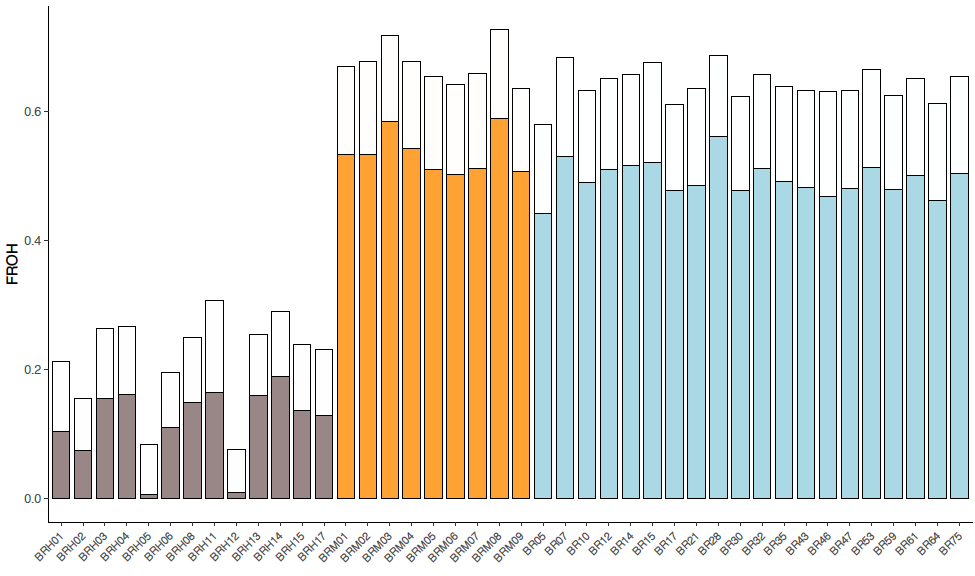


(b)


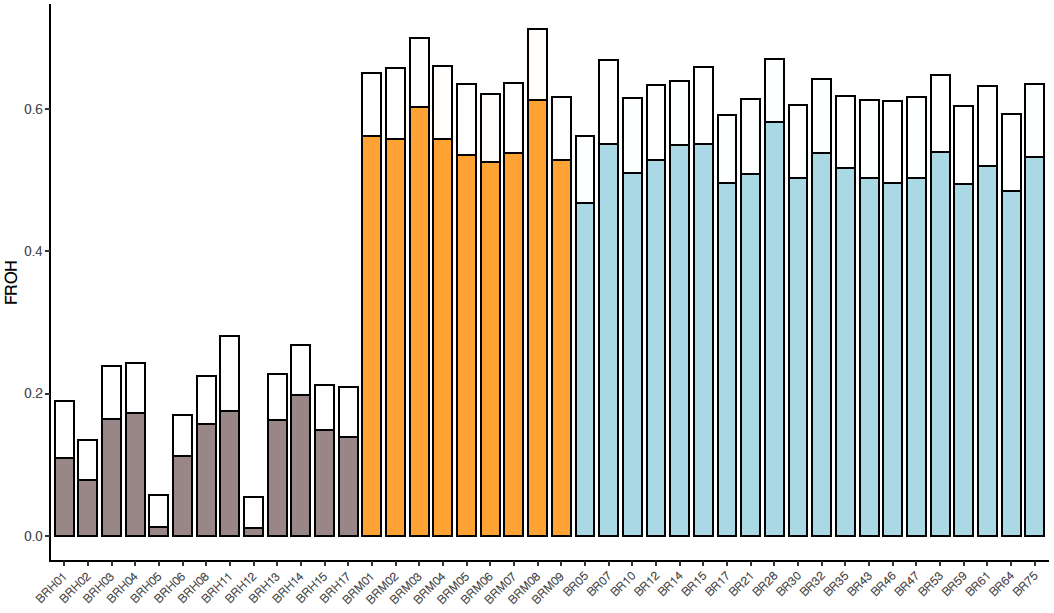


(c)


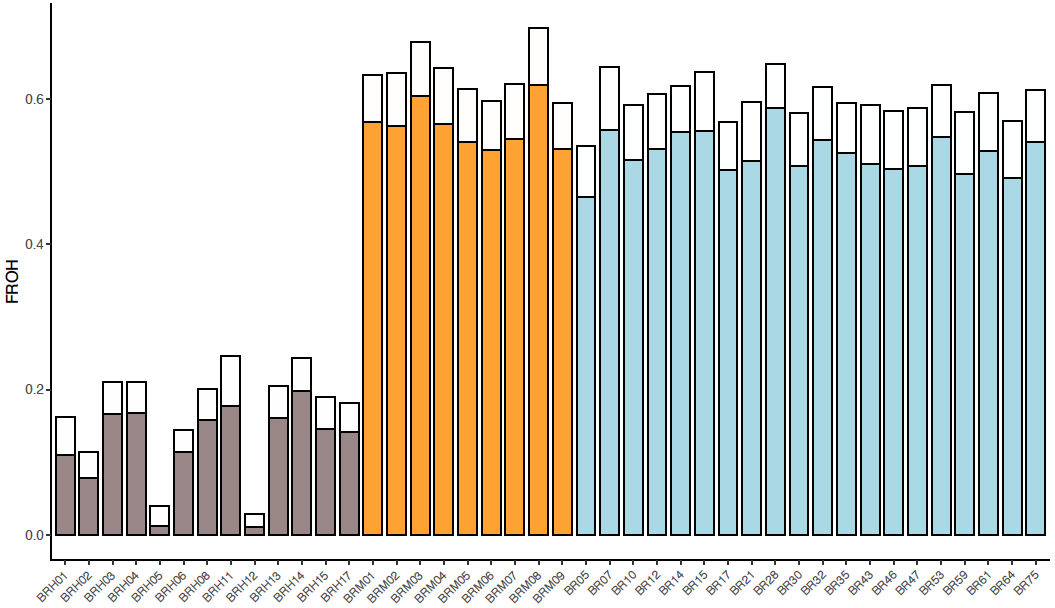


**Figure S8**. F_ROH_ for 42 genomes using **(a)** PLINK (*homozyg-window-het* 1, *homozyg-window-snp* 100), **(b)** PLINK (*homozyg-window-het* 3, *homozyg-window-snp* 250) **(c)** (*homozyg-window-het* 5, *homozyg-window-snp* 500), as shown in main text.

1. **Mutational load**


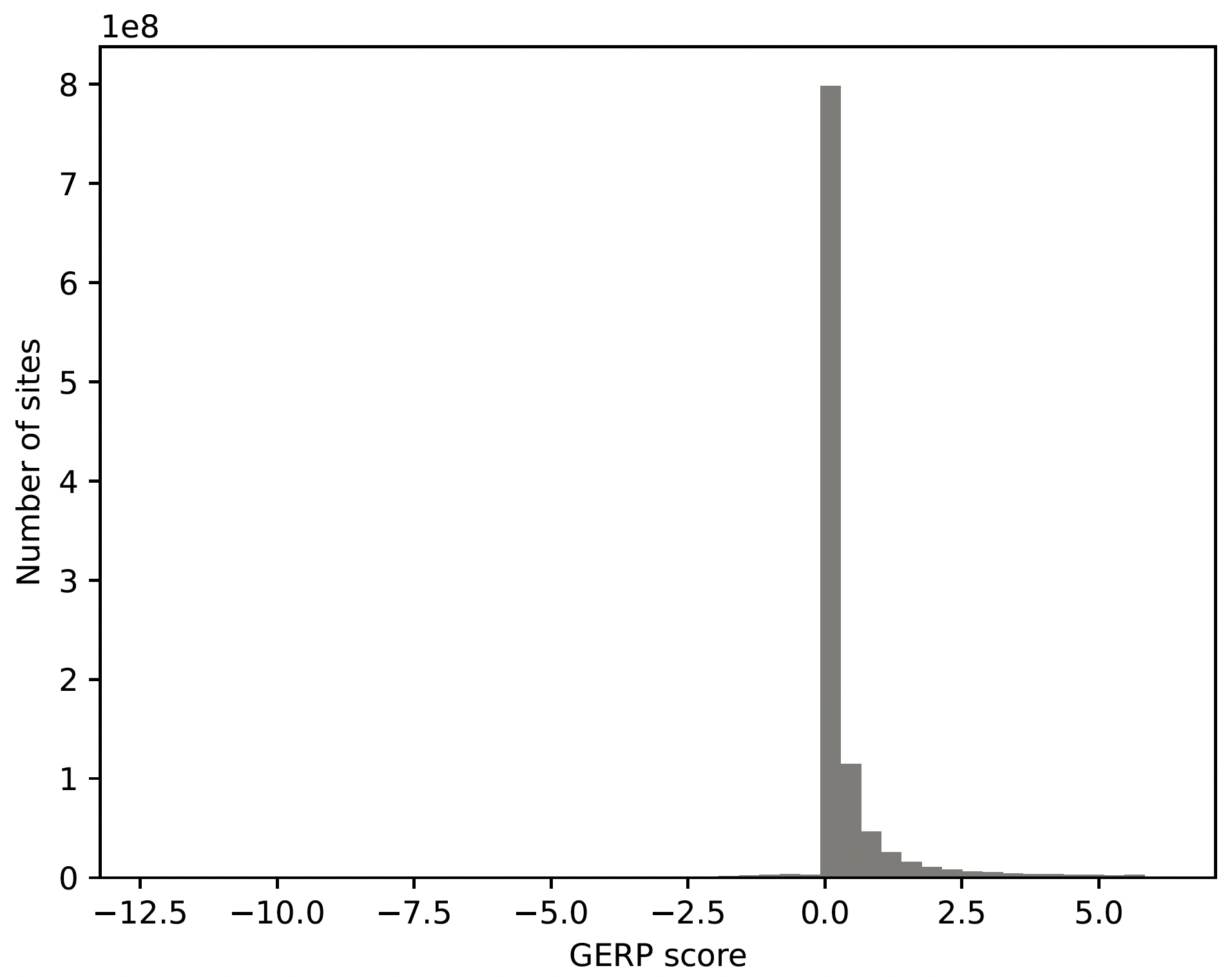


**Figure S9**. Distribution of GERP scores. The maximum GERP score was 6.08.


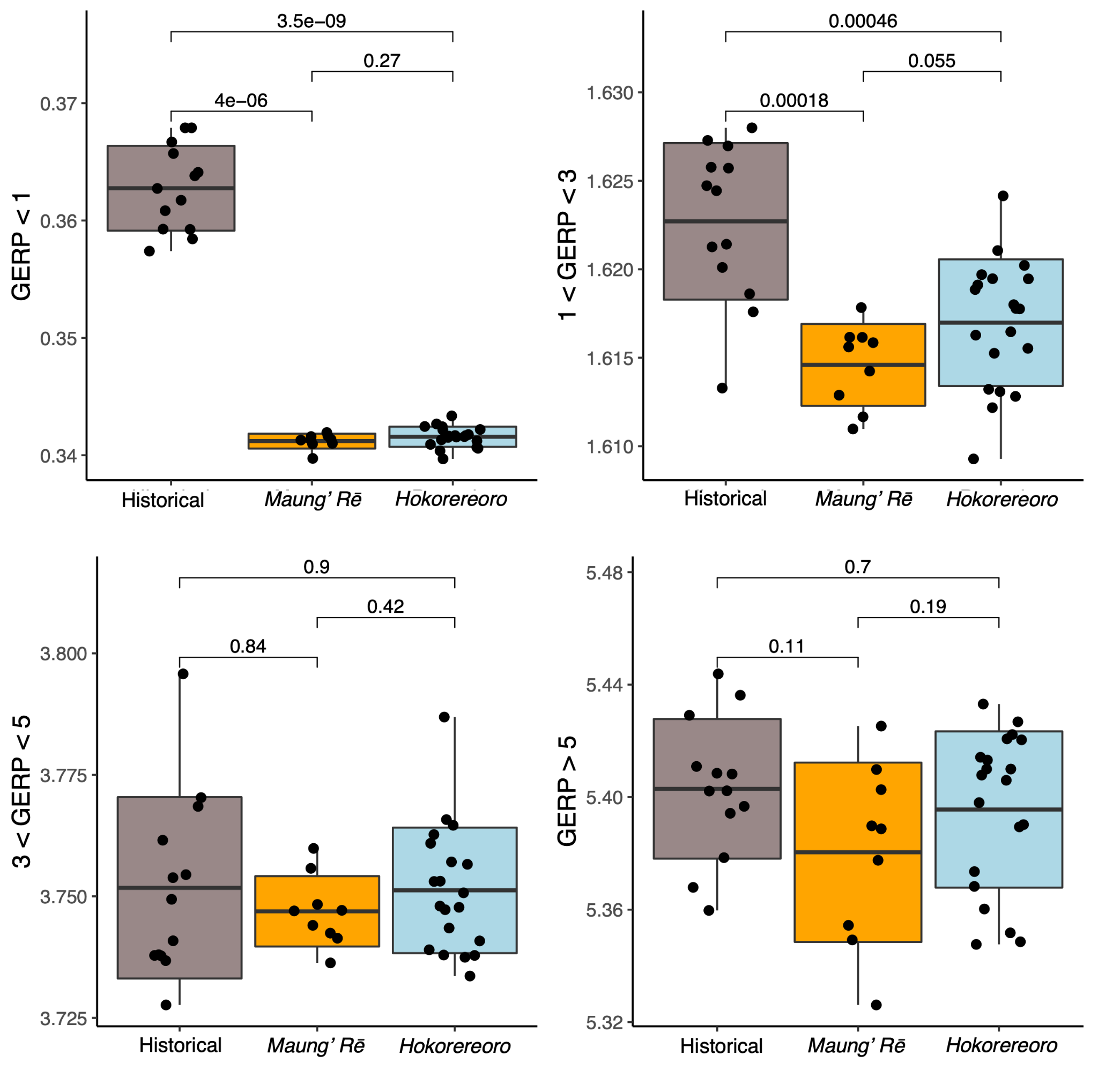


**Figure S10.** Relative individual load per category (GERP scores <1, 1-3, 3-5, and >5) in historical and modern populations. Horizontal lines within box plots depict the mean, bounds of boxes represent the standard deviation and vertical bars represent minima and maxima.


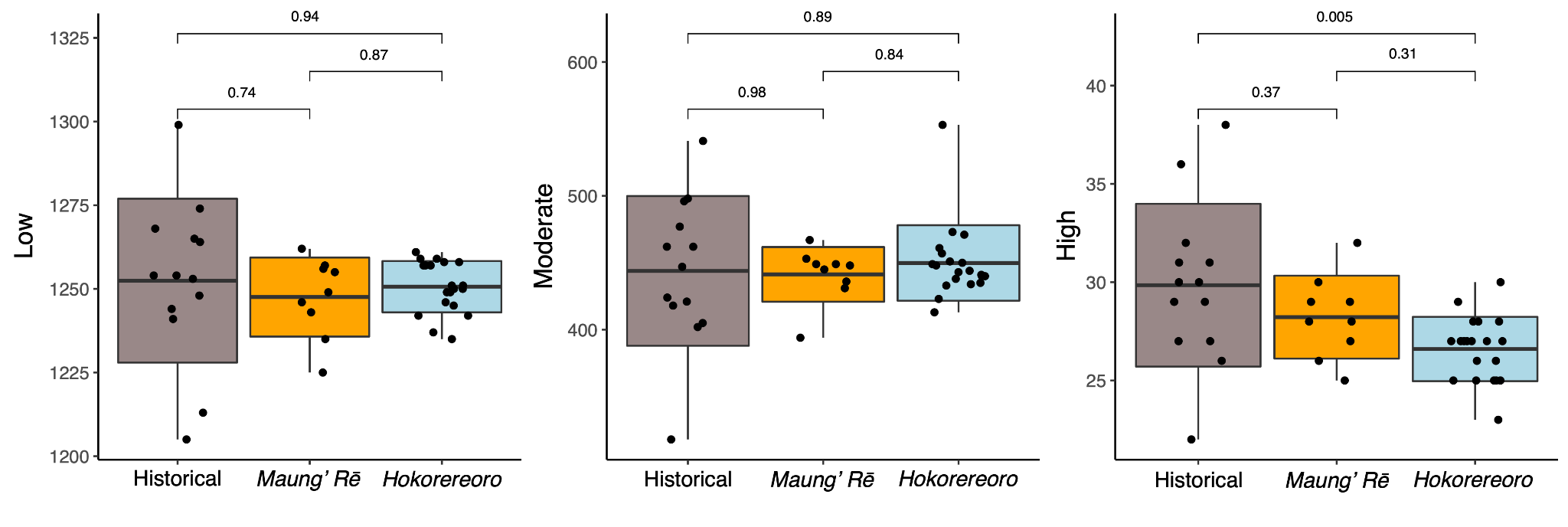


**Figure S11**. Variant counts per individual for Low impact variants (i.e., synonymous) estimated with SnpEff. Horizontal lines within box plots depict the mean, bounds of boxes represent the standard deviation and vertical bars represent minima and maxima. The lack of difference among populations supports the assumption that Low impact variants are mostly synonymous [[9]](https://paperpile.com/c/EDHKv8/nKJJ) and indicates that there is little to no bias associated with mapping genomes of either population to the reference, as shown in Fig. S3a.


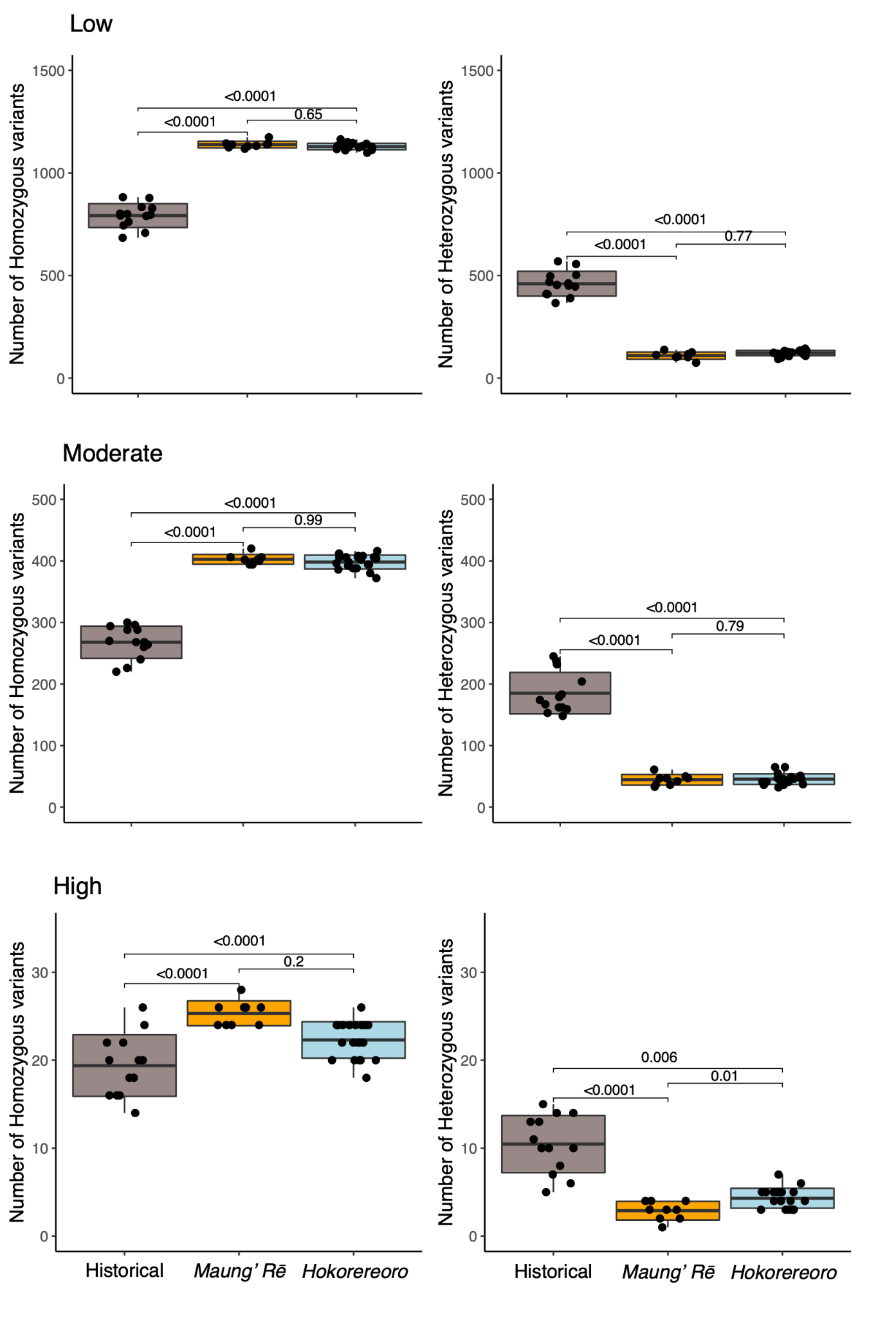


**Figure S12**. Number of variants per individual for Low, Moderate and High impact variants separated by homozygous and heterozygous state. Horizontal lines within box plots depict the mean, bounds of boxes represent the standard deviation and vertical bars represent minima and maxima.

**References**

[1. Bolger AM, Lohse M, Usadel B. Trimmomatic: a flexible trimmer for Illumina sequence data. Bioinformatics. 2014;30:2114–20.](http://paperpile.com/b/EDHKv8/1NXwy)

[2. Li H, Durbin R. Fast and accurate long-read alignment with Burrows-Wheeler transform. Bioinformatics. 2010;26:589–95.](http://paperpile.com/b/EDHKv8/hLZgd)

[3. Li H. Toward better understanding of artifacts in variant calling from high-coverage samples. Bioinformatics. 2014;30:2843–51.](http://paperpile.com/b/EDHKv8/553Zg)

[4. Li H, Handsaker B, Wysoker A, Fennell T, Ruan J, Homer N, et al. The Sequence Alignment/Map format and SAMtools. Bioinformatics. 2009;25:2078–9.](http://paperpile.com/b/EDHKv8/v7HHR)

[5. Quinlan AR, Hall IM. BEDTools: a flexible suite of utilities for comparing genomic features. Bioinformatics. 2010;26:841–2.](http://paperpile.com/b/EDHKv8/cDwdc)

[6. Garrison E, Marth G. Haplotype-based variant detection from short-read sequencing. arXiv [q-bio.GN]. 2012.](http://paperpile.com/b/EDHKv8/raZU)

[7. Danecek P, Auton A, Abecasis G, Albers CA, Banks E, DePristo MA, et al. The variant call format and VCFtools. Bioinformatics. 2011;27:2156–8.](http://paperpile.com/b/EDHKv8/UjH8Q)

[8. Kiełbasa SM, Wan R, Sato K, Horton P, Frith MC. Adaptive seeds tame genomic sequence comparison. Genome Res. 2011;21:487–93.](http://paperpile.com/b/EDHKv8/gJfu)

[9. Cingolani P, Platts A, Wang LL, Coon M, Nguyen T, Wang L, et al. A program for annotating and predicting the effects of single nucleotide polymorphisms, SnpEff: SNPs in the genome of Drosophila melanogaster strain w1118; iso-2; iso-3. Fly . 2012;6:80–92.](http://paperpile.com/b/EDHKv8/nKJJ)
